# Circular RNA circDLC1 inhibits MMP1-mediated liver cancer progression via interaction with HuR

**DOI:** 10.7150/thno.53227

**Published:** 2021-01-01

**Authors:** Hailing Liu, Tian Lan, Hui Li, Lin Xu, Xing Chen, Haotian Liao, Xiangzheng Chen, Jinpeng Du, Yunshi Cai, Jinju Wang, Xuefeng Li, Jiwei Huang, Kefei Yuan, Yong Zeng

**Affiliations:** 1 Department of Liver Surgery & Liver Transplantation, State Key Laboratory of Biotherapy and Cancer Center, West China Hospital, Sichuan University and Collaborative Innovation Center of Biotherapy, Chengdu, 610041, China.; 2 Laboratory of Liver Surgery, West China Hospital, Sichuan University, Chengdu, 610041, China.; 3 School of Basic Medical Sciences, Guangzhou Medical University, Guangzhou, 511436, China.; 4 Shenzhen Luohu People's Hospital, The Third Affiliated Hospital of Shenzhen University, Shenzhen, 518001, China.

**Keywords:** Hepatocellular carcinoma, KIAA1429, circular RNA, RNA-binding protein HuR, MMP1

## Abstract

**Rationale:** circular RNAs (circRNAs) have been demonstrated to play a crucial role in cancer progression. KIAA1429, a key component of the m6A methyltransferase complex, has recently been reported to promote hepatocellular carcinoma (HCC) progression by regulating the m6A methylation. The aim of present study is to investigate the role of circular RNAs in KIAA1429-mediated HCC progression.

**Methods:** RNA sequencing (RNA-seq) and methylated RNA immunoprecipitation sequencing (m6A-seq) were utilized to identify KIAA1429-regulated circRNAs. The effects of circDLC1 on proliferation and metastasis of hepatoma cells were examined *in vitro* and *in vivo*. RT-qPCR was used to measure the expression of circDLC1 in HCC tissues and hepatoma cells. RNA FISH, RIP assays and biotin-labeled RNA pull-down were used to investigate the downstream effector of circDLC1. The downstream targets of circDLC1 were identified using RNA-seq.

**Results:** Our data demonstrated that circDLC1 was downregulated in HCC tissues and closely relevant to favorable prognosis. Overexpression of circDLC1 inhibited the proliferation and motility of hepatoma cells *in vitro* and *in vivo,* while silencing of circDLC1 played the opposite role. Mechanistic investigations revealed that circDLC1 could bind to RNA-binding protein HuR, which subsequently reduced the interaction between HuR and MMP1 mRNAs, and thus inhibited the expression of MMP1, ultimately contributing to inhibition of HCC progression.

**Conclusion:** Our work suggests that circDLC1, a downstream target of KIAA1429, is a promising prognostic marker for HCC patients, and the circDLC1-HuR-MMP1 axis may serve as a potential therapeutic target for HCC treatment.

## Introduction

Liver cancer is one of the most common malignant tumors and the fourth leading cause of cancer-related death worldwide 1. Hepatocellular carcinoma (HCC), which is the major form of liver cancer, consists of around 90% of primary liver cancer 2. The high rate of postsurgical recurrence and metastasis is the main cause of poor prognosis in HCC patients, with a five-year survival of less than 20% 3, 4. However, the molecular mechanisms underlying HCC growth and metastasis remain largely unknown.

N6-Methyladenosine (m6A) modification, methylation of adenosine at the N6 position, is a widespread and abundant modification in messenger RNA (mRNA) and non-coding RNAs (ncRNAs) in eukaryotes 5. Emerging studies revealed that m6A modification is tightly correlated with cancer-associated signaling pathways, including RNA stability, cell fate determination, transcription splicing, translation efficiency, and drug resistance 6-8. KIAA1429, a key component of the m6A methyltransferase complex, has been proved to be crucial for RNA m6A methylation 5. A series of studies have shown that KIAA1429 is aberrantly expressed in many types of cancer, such as breast cancer 9, 10, gastric cancer 11 and HCC 12, 13 and regulates the proliferation and metastasis of cancer cells. Previous studies only focused on KIAA1429-mediated post-translational modification on mRNA. However, the modification of KIAA1429 on non-coding RNAs and its effect on downstream signaling pathway remain unknown.

Circular RNAs (circRNAs), known as covalently closed and single-stranded transcripts, have been proved to exist in many species 14. Recent studies have reported that circRNAs was aberrantly expressed in many types of cancer, such as HCC 15, gastric cancer 16, and pancreatic ductal adenocarcinoma 17. The main function of circRNAs in tumor is sponging microRNAs (miRNAs), encoding protein or binding protein 15-17. Interestingly, more recent studies revealed that methyltransferase-like-3 (METTL3), core complex of methyltransferase, is involved in circRNA biogenesis and function by regulating m6A modification 18, 19. Thus, the specific roles of KIAA1429 in mediating circRNAs pathway are urgently needed to explore.

In this study, we identified circDLC1 (derived from the exons 14, 15 and 16 of the DLC1 gene) as a KIAA1429-regulated circRNA, which was closely associated with favorable prognosis. In addition, circDLC1 was found to interact with RNA-binding protein HuR and block the interaction between HuR and MMP1 mRNAs. Our study identified a novel circRNA that was regulated by m6A modification and provided new insights into the pathogenesis of HCC.

## Materials and Methods

### Human samples

All HCC and paired adjacent normal tissue samples (n = 110) were obtained from deidentified patients who had undergone surgical resection in West China Hospital (Sichuan University, Chengdu, China). The protocols of this study were conducted in accordance with the Ethical Review Committees of Sichuan University. According to the policies of the committee, the written informed consent was provided from each patient.

### Cell lines

Huh-7, Hep3B, HepG2 and SK-Hep1 cell lines were purchased from the Shanghai Cell Bank Type Culture Collection Committee (CBTCCC, Shanghai, China). SNU449 cell line was obtained from ATCC (Manassas, VA, USA). Short tandem repeat (STR) analyses were performed by third-party biology services with all cell lines to characterize the identities of the cells (Feiouer Biology Co., Ltd., Chengdu, China). All cells were cultured in DMEM (HyClone, Logan, UT, USA) containing 10% FBS (HyClone) at 37 °C and 5% CO_2_. Actinomycin D was purchased from Sigma-Aldrich.

### RNA fluorescence *in situ* hybridization (FISH)

Cy3-labled probes against 18S and U6 were designed and synthesized by RiboBio (RiboBio Biotechnology). Cy3-labelled circDLC1 probes (Supplementary table S6) were synthesized by Sangon Biotech. RNA FISH was conducted using the Fluorescent *in situ* Hybridization Kit (RiboBio Biotechnology), according to the manufacturer's instruction. Images were acquired by the A1RþMP Confocal Laser Microscope System (Nikon).

### Cross-Linked RNA immunoprecipitation assays

0.3% formaldehyde was used to cross-link and glycine solution was used to quench the cells (Millipore). Cross-Linked RNA immunoprecipitation assays were performed according to the manufacturer's protocol of Magna RIP^TM^ RNA-binding Protein Immunoprecipitation Kit (Millipore, Massachusetts, USA). In brief, magnetic beads were incubated with 5 μg of antibody against HuR (Abcam, Cambridge, USA) or DHX9 (Abcam, Cambridge, USA) and normal IgG (Millipore, Massachusetts, USA) for 30 min at 25 °C. The cell lysate and treated magnetic beads were incubated overnight at 4 °C. After 6 times washing, proteinase K digestion buffer was used to digest the RNA-protein complexes for 45 min at 55 °C. The enriched RNAs were extracted by phenol: chloroform: isoamyl alcohol (125 : 24 : 1). Then, the purified RNAs were subsequently analyzed by RT-qPCR to assess the enrichment of target RNAs to the target proteins.

### Biotin-labeled RNA pull-down

0.3% formaldehyde was used to cross-link and glycine solution was used to quench the cells (Millipore). Sangon Biotech synthesized the biotin-labelled circDLC1 probes and control probes (Table S6). Biotin-labeled RNA pull-down was conducted according to the manufacturer's instructions of the EZ-Magna ChIRP RNA Interactome Kit (Millipore). Briefly, 1/5 associated RNA-protein complexes were separated for RNA purification, and the remaining were subjected to protein purification. RNA was purified with RNeasy Mini Kit (Qiagen Inc.). RT-qPCR was used to identify and quantify the enrichment of circDLC1. On the other hand, 4/5 associated RNA-protein complexes were treated with RNase A, RNase H and DNase to elute proteins. The purified proteins were subsequently analyzed by Western blot.

### Animal studies

All animal experiments were authorized by the Institutional Animal Care and Use Committee of Sichuan University. In this study, the BALB/c nude mice were all male and 6 weeks old, which were purchased from BEIJING HFK BIOSCIENCE (Beijing, China). All mice were fed under standard pathogen-free conditions. All surgical procedures were performed with sodium pentobarbital anesthesia. 5 × 10^5^ cells were injected subcutaneously into the right axilla of mice. Tumor volume was measured by a caliper weekly and calculated as length × width^2^ × 0.52. For the liver orthotopic-implanted models, each liver of mice was injected with 1 × 10^6^ cells. After 6 weeks, mice were sacrificed, and the livers were visualized using the IVIS@ Lumina II system (Caliper Life Sciences, Hopkinton, MA). All tumor samples were embedded in paraffin and confirmed by hematoxylin and eosin staining. Furthermore, 2 × 10^6^ cells were injected into mice to established the tail intravenous injection models through the tail vein. After 6 weeks, mice were sacrificed, and the lungs were visualized using the IVIS@ Lumina II system (Caliper Life Sciences, Hopkinton, MA).

### Statistical analysis

All statistical analyses were performed using GraphPad Prism 8 software (GraphPad Software). The Student *t* test, ×2 test, the Wilcoxon signed-rank test, was conducted as appropriate. Kaplan-Meier method was applied to measure the survival curves, and the log-rank test was used to assess the differences. Correlations were assessed by Pearson correlation analysis. *P* values less than 0.05 indicated statistical significance. *P < 0.05, **P < 0.01, ***P < 0.001.

## Results

### CircDLC1 is a KIAA1429-regulated circRNA and clinically relevant to HCC patient prognosis

**Figure 1:**
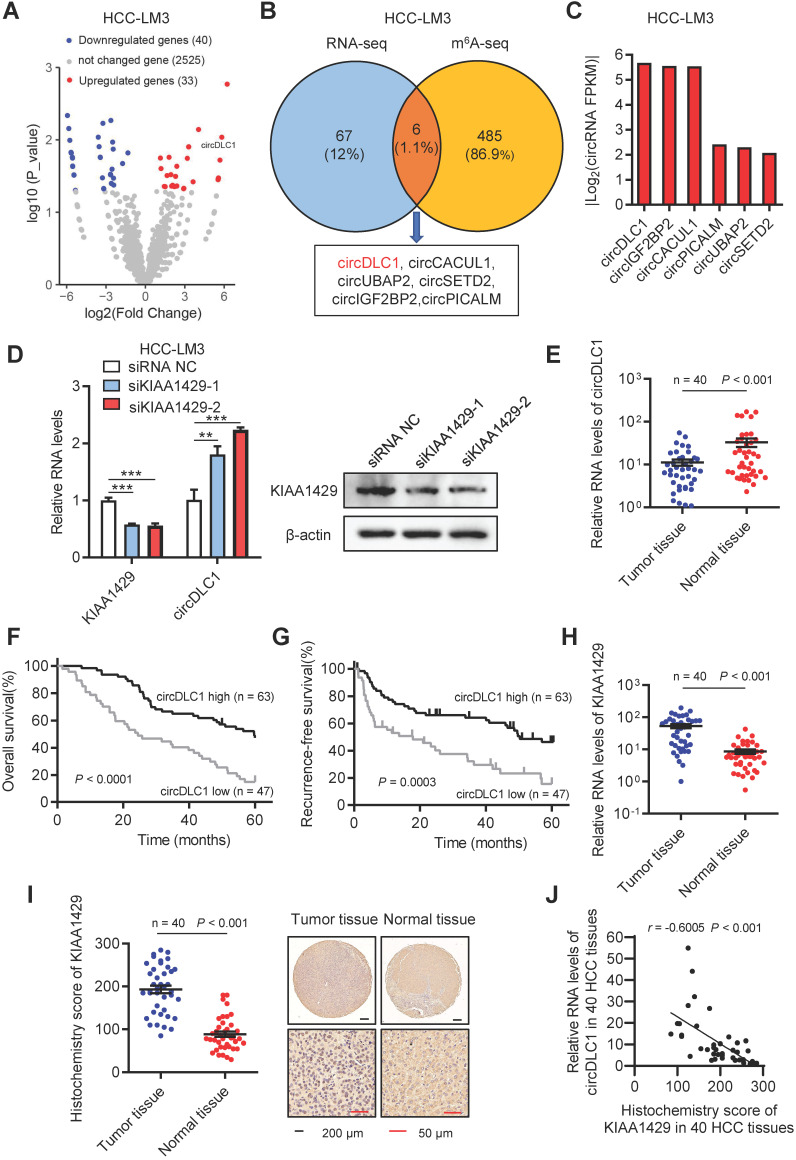
** CircDLC1 is a KIAA1429-regulated circRNA and closely relevant to patient prognosis. (A)** Volcano plot of the differentially expressed circRNAs between shKIAA1429 and shCtrl cells. **(B)** Six circRNAs were identified through the intersection of circRNA-analysis and m^6^A-seq. **(C)** The fold change of candidate circRNAs caused by KIAA1429 depletion in circRNA-analysis dataset. **(D)** CircDLC1 expression in KIAA1429 knockdown cells by using RT-qPCR. Data are presented as mean ± SD, student's t-test was used. **(E)** circDLC1 expression in 40 pairs of HCC tissues and adjacent normal tissues by using RT-qPCR. The data are presented as mean ± SEM, Wilcoxon signed-rank test was used. **(F) and (G)** Kaplan-Meier's survival curves showed the correlations between circDLC1 expression and OS or RFS. Log-rank test was used. **(H)** The relative mRNA levels of KIAA1429 expression in 40 pairs of HCC tissues and adjacent normal tissues by using RT-qPCR. The data are presented as mean ± SEM, Wilcoxon signed-rank test was used. **(I)** IHC stains of KIAA1429. (Left) Histochemistry score of KIAA1429 in 40 HCC tissues and adjacent normal tissues. (Right) Representative samples. The data are presented as mean ± SEM, Wilcoxon signed-rank test was used.** (J)** The correlation between the IHC stains of KIAA1429 and the relative expression of circDLC1 in 40 HCC tissues. The correlation was measured by Pearson correlation analysis. OS, overall survival; RFS, recurrence-free survival; IHC, immunohistochemistry; RT-qPCR, quantitative reverse transcription PCR; **P < 0.01, ***P < 0.001.

To identify circRNAs that are regulated by KIAA1429, we first characterized circular RNA transcripts using RNA-seq from KIAA1429 stable knockdown cells (shKIAA1429) and control cells (shCtrl). We detected 73 distinct circRNAs with 2-fold change (*p*<0.05). Among these differentially expressed circRNAs, 33 were upregulated and 40 were downregulated in shKIAA1429 cells compared with shCtrl cells (Figure 1A). Our previous work has revealed the target transcripts of KIAA1429-mediated m6A methylation by m6A-seq 13. To identify the circRNAs directly regulated by KIAA1429, we take intersection of RNA-seq and m6A-seq, 6 circRNAs (circDLC1, circCACUL1, circUBAP2, circSETD2, circIGF2BP2, circPICALM) were screened out (Figure 1B). Among them, the expression of circDLC1 showed the most remarkable change upon KIAA1429 silencing (Figure 1C), indicating that circDLC1 could be the main downstream target of KIAA1429. To verify the RNA-seq results, the expression of circDLC1 in shKIAA1429 and shCtrl hepatoma cells was examined by RT-qPCR. As shown in Figure 1D, the expression of circDLC1 was significantly increased in HCC-LM3 cells transfected with two independent KIAA1429 siRNAs (siKIAA1429-1 and siKIAA1429-2) compared to cells transfected with negative control (NC) siRNA.

Next, we examined the expression of circDLC1 in paired HCC and adjacent normal tissue samples (n = 40) by RT-qPCR. The expression of circDLC1 was much lower in HCC tissues than paired adjacent normal tissues (Figure 1E and Figure S1A). Furthermore, to investigate the correlation between the expression level of circDLC1 and HCC patient prognosis, the expression levels of circDLC1 in 110 HCC patients (including the previous 40 samples) were measured. According to the relative expression level of circDLC1, the patients were divided into circDLC1 high expression group (n = 63) and circDLC1 low expression group (n = 47). It was revealed that circDLC1 low expression group showed more advanced tumor stage (Table S1), demonstrating that circDLC1 was associated with HCC progression. In univariate analysis, we found that the status of ascites, serum AFP level, TNM stage and BCLC stage, microvascular invasion, macrovascular invasion and circDLC1 expression were correlated with OS or RFS (Table S2). In multivariate regression analysis, the status of ascites, microvascular invasion and low circDLC1 expression were shown to be independent risk factors for OS, while serum AFP level and low circDLC1 expression were independent risk factors for RFS (Table S3). In addition, according to the Kaplan-Meier survival analyses, low expression of circDLC1 was associated with worse OS (*P* < 0.0001) and RFS (*P* = 0.0003) (Figure 1F, G).

On the other hand, the expression of KIAA1429 in paired HCC and adjacent normal tissue samples (n = 40) was examined by RT-qPCR and IHC. Notably, the expression of KIAA1429 was obviously upregulated in HCC tissues compared to paired adjacent normal tissues (Figure 1H, I). Moreover, we revealed a negative correlation between the expression of KIAA1429 and circDLC1 in HCC tissues (Figure 1J). Furthermore, FISH and immunofluorescent assays at a subcellular level confirmed the negative correlation between KIAA1429 and circDLC1 (Figure S1C). Collectively, these results indicated that circDLC1 was regulated by KIAA1429 and low circDLC1 expression predicted poor prognosis in HCC patients.

### The characteristics of circDLC1

**Figure 2:**
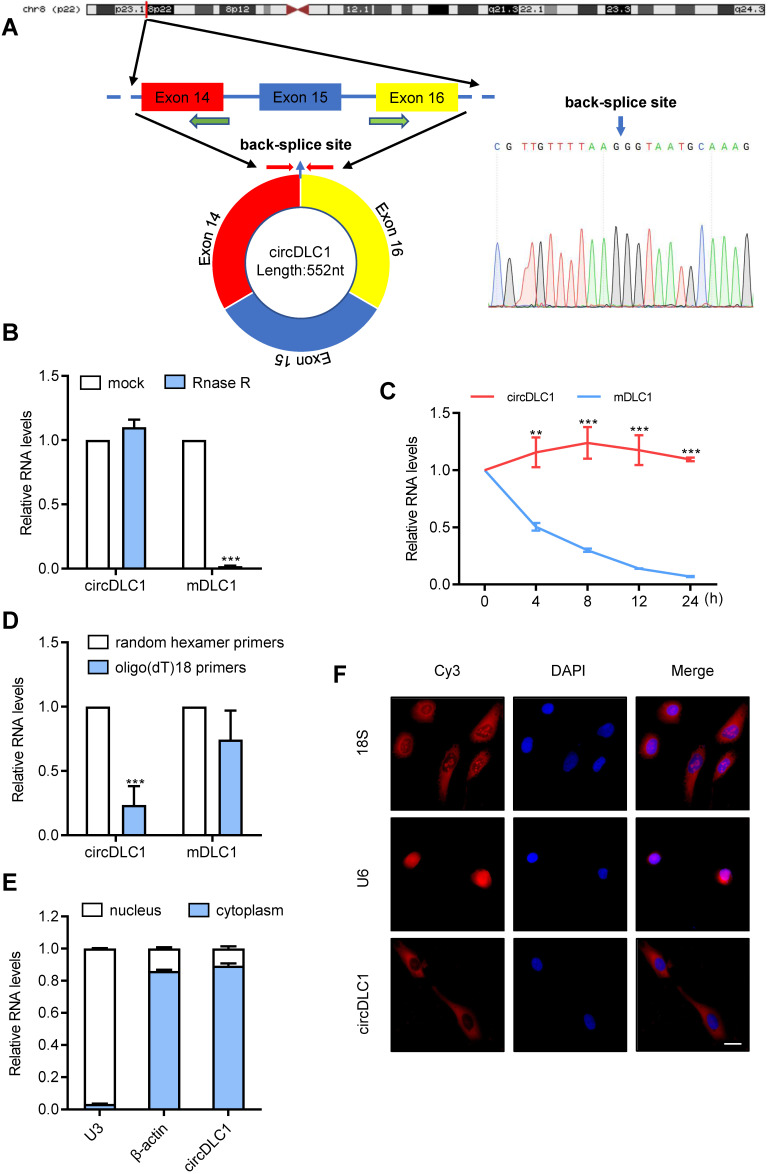
** Characterization of circDLC1. (A)** Scheme illustrating the production of circDLC1. PCR primers used to specifically detect circDLC1 by RT-qPCR are indicated by red arrows. **(B)** The relative RNA levels were analyzed by RT-qPCR and normalized to the value detected in the mock group. **(C)** The relative RNA levels of circDLC1 and Mdlc1 were analyzed by RT-qPCR after treatment with Actinomycin D at the indicated time points. **(D)** Random hexamer or oligo (dT)18 primers were used in the reverse transcription experiments. The relative RNA levels were analyzed by RT-qPCR and normalized to the value using random hexamer primers. **(E)** circDLC1 are abundant in the cytoplasm of SNU 449 cells, β-actin and U3 were applied as positive controls in the cytoplasm and nucleus, respectively.** (F)** RNA FISH for circDLC1. Nuclei were stained with DAPI. 18S and U6 were applied as positive controls in the cytoplasm and nucleus, respectively. Scale bar, 50 µm. For (B), (C) and (D), data are presented as mean ± SD. Student's t test was used. ***P < 0.001.

Next, we examined the physical circular structure of circDLC1. CircDLC1 is derived from exons 14, 15 and 16 of the DLC1 gene [CircBase ID: hsa_circ_0135718, termed circDLC1], and the result of Sanger sequencing proved the presence of back-splicing junction (Figure 2A). Also, circDLC1 was observed to resist the digestion by RNase R that specifically degraded linear RNAs but not circRNAs (Figure 2B). Owing to its circular structure, circDLC1 was more stable than mDLC1 with actinomycin D treatment (Figure 2C). Moreover, random hexamer and oligo (dT)18 primers were utilized to further confirm the circularity of circDLC1, circDLC1 was reduced in reverse-transcription efficiency by oligo-dT primers due to the lack of polyA tail (Figure 2D). These results indicated that the formation of circDLC1 was not due to genomic rearrangement. To investigate the localization of circDLC1, we performed subcellular RNA fractionation assays and FISH, which demonstrated the cytoplasmic enrichment of circDLC1 (Figure 2E, F). Collectively, these findings demonstrated the circularity of circDLC1 and the localization of circDLC1 in hepatoma cells.

### DHX9 can regulate the expression of circDLC1

**Figure 3:**
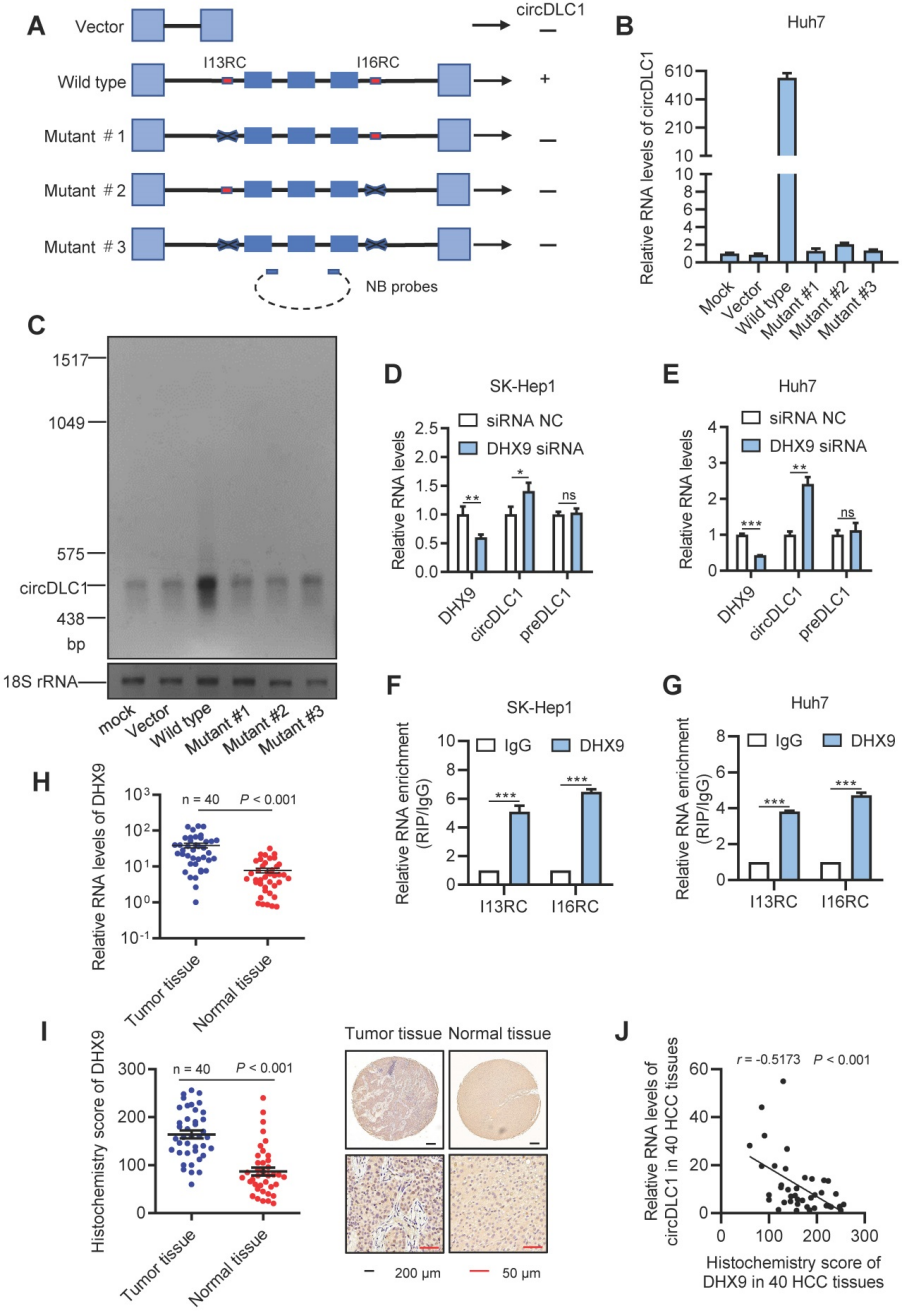
** DHX9 can regulate the expression of circDLC1. (A)** A schematic drawing of four types of circDLC1-overexpressing vectors. The genomic region for circDLC1 (blue bars) with its wild-type flanking introns (black lines) was inserted into the pEZ expression vector (wild type). I13RC and I16RC are indicated by red bars. GFP sequences from the expression vector backbone are indicated by light blue bars. A series of deletions are indicated by black crosses (mutant#1 to mutant#3). Northern blot probes targeting circDLC1 are indicated by blue bars with dotted lines. **(B) and (C)** RT-qPCR and Northern blot showed the expression of circDLC1 after transfection with the four types of circDLC1-overexpressing vectors (wild type, mutant#1 to mutant#3). **(D) and (E)** RT-qPCR for circDLC1, and preDLC1 upon DHX9 depletion using RNAi in Hepatoma cell lines. **(F) and (G)** RIP experiments were performed using an Ab against DHX9 on extracts from hepatoma cells. **(H)** The relative mRNA levels of DHX9 in 40 paired HCC tissues and adjacent normal tissues by using RT-qPCR. **(I)** IHC stains of DHX9. (Left) Histochemistry score of DHX9 in 40 HCC tissues and adjacent normal tissues. (Right) Representative samples. **(J)** The correlation between the IHC stains of DHX9 and the relative expression of circDLC1 in 40 HCC tissues. The correlation was measured by Pearson correlation analysis. For (B, D-G), Data are presented as mean ± SD; n = 3. Student's t test was used. *P < 0.05, **P < 0.01, ***P < 0.001.

Previous study revealed that the long flanking introns with inverted complementary sequences usually facilitated the biogenesis of circRNAs from their internal exons 20, 21. These complementary sequences can promote the biogenesis of circRNAs through enhancing back-splicing. By comparing the sequence of flanking introns of the circDLC1 gene, the highly reverse complementary sequences were observed between intron 13 and intron 16 (77% identity over 230 nucleotides, Figure S1B). They were named as I13RC (reverse complementary sequences in intron 13) and I16RC (reverse complementary sequences in intron 16), respectively. Next, to confirm whether the biogenesis of circDLC1 was determined by I13RC and I16RC, we constructed a series of plasmids of circDLC1, including wild type (a 3754-nt region of the DLC1 gene, spanning from intron 13 to intron 16), mutant #1 (deletion of I13RC), mutant #2 (deletion of I16RC) and mutant #3 (deletion of I13RC and I16RC) (Figure 3A). The result of RT-qPCR indicated that only the wild type plasmid could overexpress circDLC1 after transfection with five types of plasmids, which demonstrated that I13RC and I16RC were essential for the production of circDLC1 (Figure 3B). In addition, northern blot further confirmed this result (Figure 3C).

Previous studies have demonstrated that DHX9 could bind to the flanking inverted complementary sequences of its target RNAs to inhibit the pairing of these sequences 22, 23. To test whether DHX9 regulate the expression of circDLC1, we examined the expression of circDLC1 in hepatoma cell lines after silencing DHX9. In DHX9 knockdown cells, the expression of circDLC1 was obviously increased, while preDLC1 did not show significant changes (Figure 3D, E and Figure S1D). Moreover, RNA immunoprecipitation assays (RIP) demonstrated that the I13RC and I16RC were significantly enriched in DHX9 immunoprecipitates (Figure 3F, G). Also, we measured the expression of DHX9 in HCC samples (n = 40). DHX9 was remarkably upregulated in HCC tissues (Figure 3H, I) and the expression of circDLC1 and DHX9 showed negative correlation in HCC tissues (Figure 3J). Importantly, FISH and immunofluorescent assays at a subcellular level further confirmed the negative correlation between DHX9 and circDLC1 (Figure S1E). Collectively, our data indicated that DHX9 could bind to the flanking inverted complementary sequences of circDLC1 and inhibit the pairing of these sequences, which subsequently inhibited the production of circDLC1.

### CircDLC1 inhibits proliferation and metastasis of hepatoma cells *in vitro* and *in vivo*

**Figure 4:**
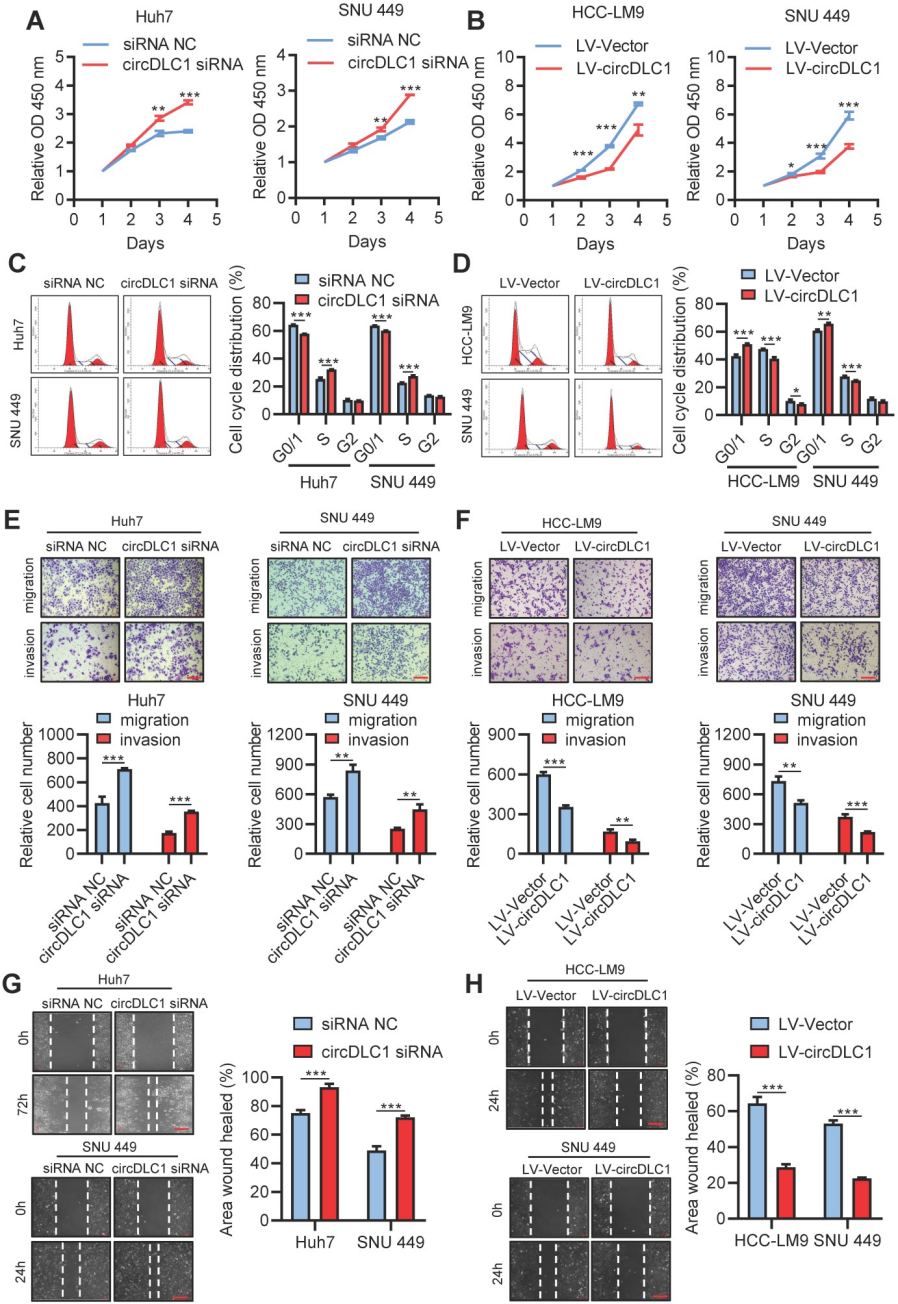
** circDLC1 inhibit hepatoma cells proliferation, migration and invasion *in vitro*. (A)** Cell Counting Kit-8 assays were performed to assess cell growth for Huh7 and SNU449 cells transfected with circDLC1 siRNA or siRNA NC. **(B)** Cell Counting Kit-8 assays were performed to assess cell growth for HCC-LM9 and SNU449 cells transfected with LV-circDLC1 or LV-vector.** (C)** Cell-cycle distribution was measured by propidium iodide staining in Huh7 and SNU449 cells transfected with circDLC1 siRNA or siRNA NC, followed by flow cytometric analysis. **(D)** Cell-cycle distribution was measured by propidium iodide staining in HCC-LM9 and SNU449 cells transfected with LV-circDLC1 or LV-vector, followed by flow cytometric analysis. **(E)** Transwell migration and invasion assays for Huh7 and SNU449 cells transfected with circDLC1 siRNA or siRNA NC.** (F)** Transwell migration and invasion assays for HCC-LM9 and SNU449 cells transfected with LV-circDLC1 or LV-vector.** (G)** Scratch wound healing assays for Huh7 and SNU449 cells transfected with circDLC1 siRNA or siRNA NC. **(H)** Scratch wound healing assays for HCC-LM9 and SNU449 cells transfected with LV-circDLC1 or LV-vector. Scale bars = 200 µm. Data are presented as mean ± SD. **P < 0.01, ***P < 0.001.

Six human hepatoma cell lines were chosen to measure the expression of circDLC1 by RT-qPCR. Among them, the lowest expression of circDLC1 was observed in HCC-LM9, moderate expression of circDLC1 was observed in SNU 449, HepG2 and Hep3B, and the highest expression of circDLC1 was observed in Huh7 and SK-Hep1 (Figure S2A). For functional studies, small interfering RNAs (siRNAs) that target the back-splice sequence of circDLC1 was used to knock down the expression of circDLC1 in Huh7 and SNU 449 cells, while no significant change was found in mDLC1 and DLC1 protein levels upon circDLC1 knockdown (Figure S2B, C). Furthermore, we successfully constructed stable circDLC1-overexpressing cells with HCC-LM9 and SNU 449 cells and stable circDLC1 knockdown cells with SK-Hep1 cells (Figure S2D-F). It was shown that knockdown of circDLC1 significantly promoted cell proliferation, whereas overexpression of circDLC1 inhibited cell proliferation (Figure 4A, B). Besides, we investigated the cell cycle distribution using flow cytometry. Knockdown of circDLC1 in hepatoma cells significantly decreased the percentage of G0/G1 phase and increased the percentage in S phase, whereas the ectopic expression of circDLC1 significantly increased the percentage of G0/G1 phase and decreased the percentage in S phase (Figure 4C, D), indicating that circDLC1 can block the cell cycle. Furthermore, we investigated whether circDLC1 could affect the migration and invasion of hepatoma cells. Transwell assays with or without Matrigel showed that silencing of circDLC1 enhanced the migratory and invasive capacities of hepatoma cells, while the motility of hepatoma cells was markedly impaired when circDLC1 was overexpressed (Figure 4E, F). Moreover, scratch wound healing assays showed that knocking down circDLC1 promoted hepatoma cell migration (Figure 4G). In contrast, circDLC1 overexpression repressed hepatoma cell migration (Figure 4H).

**Figure 5:**
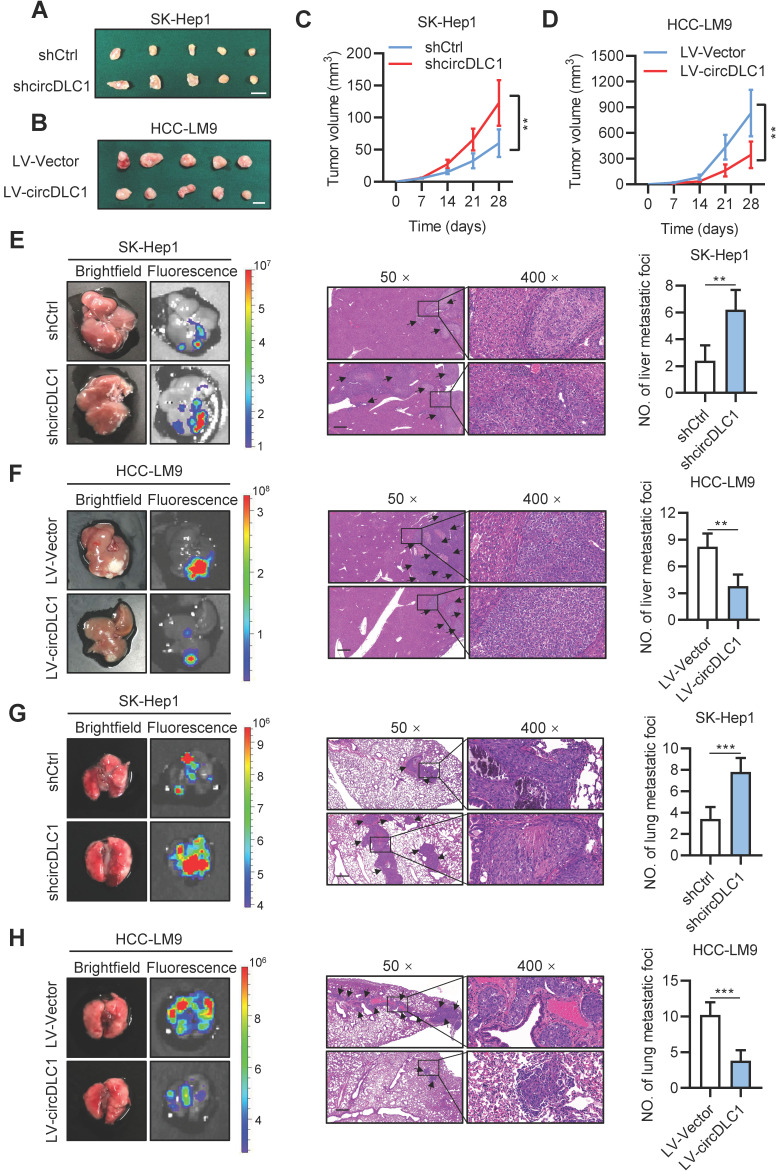
** circDLC1 inhibits tumor growth and metastasis *in vivo*. (A), (B), (C) and (D)** Tumor volume in subcutaneous xenografts models with indicated SK-Hep1 and HCC-LM9 cells. Scale bars = 10 mm. **(E)** and** (F)** Representative image (brightfield, fluorescence and HE) of intrahepatic metastatic nodules in orthotopic implantation models with indicated SK-Hep1 and HCC-LM9 cells. **(G)** and** (H)** Representative image (brightfield, fluorescence and HE) of pulmonary metastatic nodules in lung metastasis models with indicated SK-Hep1 and HCC-LM9 cells. For (E)-(H), the microscopic views of HE, Scale bars = 50 µm; Data are presented as mean ± SD. **P < 0.01, ***P < 0.001.

To further explore the effects of circDLC1 *in vivo*, circDLC1 knockdown SK-Hep1 cells and circDLC1-overexpressing HCC-LM9 cells were subcutaneously injected into nude mice. We observed that tumor volume was remarkably increased when circDLC1 was knocked down, whereas overexpression of circDLC1 showed an inverse result (Figure 5A-D). Next, the effect of circDLC1 on tumor metastasis *in vivo* was assessed by establishing liver orthotopic implanted models and tail intravenous injection models. Six weeks later, circDLC1 knockdown group showed higher fluorescence value of GFP than control group both in liver and lung. Haematoxylin eosin (H&E) staining validated the number of metastatic foci was also increased both in liver and lung. However, the liver and lung metastasis model exhibited opposite results when circDLC1 was overexpressed (Figure 5E-H). Collectively, these observations indicated that circDLC1 was a tumor suppressor, which inhibited proliferation and metastasis of hepatoma cells *in vitro* and *in vivo*.

### CircDLC1 interacts with RNA-binding protein HuR

**Figure 6:**
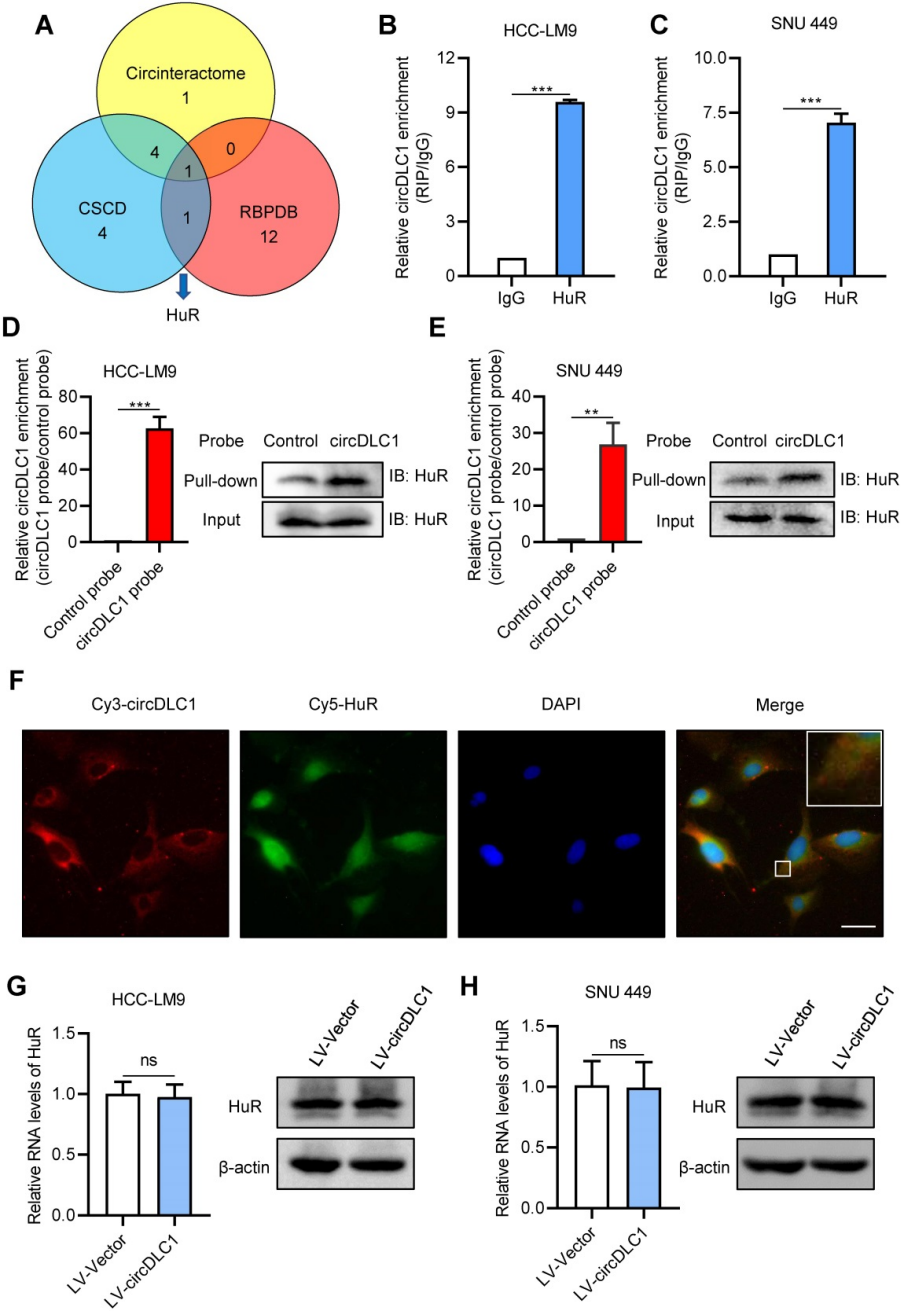
** circDLC1 interacts with RNA-binding protein HuR. (A)** HuR was identified to possibly interact with HuR through the intersection of circintractome, CSCD, and RPBDP databases.** (B)** and** (C)** RIP-qPCR analysis of the enrichment of circDLC1 on HuR relative to IgG in circDLC1-overexpressing cells. **(D)** and** (E)** Biotin-labeled RNA pull-down was performed in circDCL1-overexpressing cells using a circDLC1-specific probe and control probe, respectively. The enrichment of circDLC1 was detected by RT-qPCR and normalized to the control probe; Western blot analysis of HuR was pulled down by circDLC1-specific probe and control probe.** (F)** Co-localization between circDLC1 and HuR was observed by FISH and IF SNU 449 cells. Nuclei were stained with DAPI. Scale bar, 20 µm. **(G)** and** (H)** HuR expression was detected by RT-qPCR and western blot in circDLC1-overexpressing cells, respectively. Data are presented as mean ± SD; n = 3. Student's t test was used. ns: not significant; **P < 0.01, ***P < 0.001.

Many studies have demonstrated that circRNAs play regulatory roles via sponging miRNAs and binding proteins 16, 17, 23, 24. To confirm whether circDLC1 serves as “miRNA sponges”, RNA immunoprecipitation assays (RIP) were performed in SNU 449 and HCC-LM9 cells using a specific antibody against argonaute 2 (AGO2). The result showed that there was no significant difference between IgG group and AGO2 group, which indicated that circDLC1 may not act as “miRNA sponges” (Figure S3A). Next, we conducted bioinformatics analysis to screen the possible binding protein for circDLC1 by using circinteractome 25, CSCD 26 and RBPDB 27. The intersection of these databases suggested RNA-binding protein HuR is the most possible binding protein for circDLC1 (Figure 6A).

To confirm the interaction between circDLC1 and HuR, we then examined the subcellular location of HuR. Subcellular protein fractionation assay revealed that HuR existed in both cytoplasm and nucleus (Figure S3B). Given that circDLC1 was located in the cytoplasm, RIP assays were conducted using the anti-HuR antibody to verify whether circDLC1 could bind HuR. In HCC-LM9 and SNU 449 cells, the significant enrichment of circDLC1 was observed in HuR immunoprecipitates compared with IgG pellet (Figure 6B, C). Furthermore, we conducted biotin-labeled RNA pull-down using specific biotin-labeled circDLC1 probe (against the back-splice sequence) and control probe. The data showed that circDLC1 and HuR were prominently enriched in circDLC1 probe group in HCC-LM9 and SNU 449 cells (Figure 6D, E). Also, fluorescence *in situ* hybridization at a subcellular level confirmed interaction between circDLC1 and HuR (Figure 6F). Then we observed that overexpression of circDLC1 did not affect the HuR level significantly (Figure 6G, H). Taken together, our data demonstrated that circDLC1 may physically bind HuR without affecting HuR expression.

### CircDLC1 inhibited the expression of MMP1 by reducing the stability of MMP1 mRNAs

**Figure 7:**
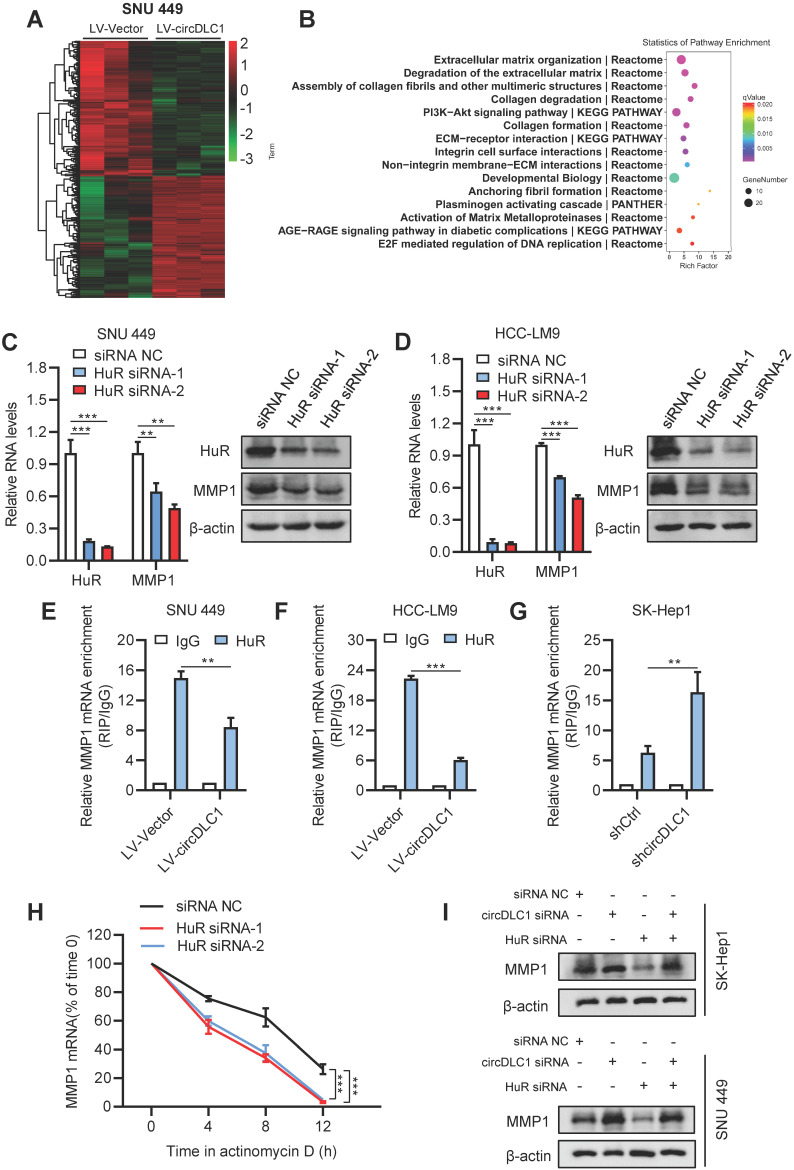
** circDLC1 inhibited the expression of MMP1 by reducing the interaction between HuR and MMP1 mRNAs. (A)** Clustered heatmap of significant differentially expressed gene in SNU 449 cells with stable circDLC1 overexpression or vector plasmid. Each sample contained a mixture of three repeats. **(B)** Pathway enrichment analysis is shown, significantly enriched pathway nominated by the gene ontology term are ranked based on the group enrichment scores. **(C) and (D)** HuR and MMP1 expressions were detected by RT-qPCR and western blot in transient HuR knockdown cells, respectively. **(E) and (F)** RIP-qPCR analysis of the enrichment of MMP1 mRNA on HuR relative to IgG in control and circDLC1-overexpressing cells.** (G)** RIP-qPCR analysis of the enrichment of MMP1 mRNA on HuR relative to IgG in shCtrl and shcircDLC1 cells.** (H)** 48 hours after transfection with siRNA NC and HuR siRNAs. MMP1 mRNA levels were examined at different times after administration of actinomycin D.** (I)** Western blot analysis of MMP1 expressions in SK-Hep1 and SNU 449 cells co-transfected with circDLC1 siRNAs, HuR siRNAs or the control. Data are presented as mean ± SD; n = 3. Student's t test was used. **P < 0.01, ***P < 0.001.

To better understand the tumor suppressor roles of circDLC1 on hepatoma cells, we performed RNA-sequencing to analyze the differentially expressed genes which affected by overexpressing circDLC1. Hierarchical clustering showed a total of 347 upregulated genes and 277 downregulated genes in circDLC1-overexpressing SNU 449 cells (Figure 7A). Pathway enrichment analysis and Gene Ontology (GO) analysis indicated that the extracellular matrix related pathways and genes showed the most significant changes (Figure 7B and Figure S3C). Among the extracellular matrix-related genes, a series of matrix metallopeptidases (MMP1, MMP2, MMP3, MMP10) exhibited remarkable reduction. Then we examined the mRNA levels of MMP1, MMP2, MMP3 and MMP10 in circDLC1-overexpressing cells and circDLC1-knockdown cells, respectively. The mRNA levels of MMP1, MMP3 and MMP10 showed significant reduction in circDLC1-overexpressing cells (Figure S3D). While in circDLC1-knockdown cells, the mRNA levels of MMP1, MMP2, MMP3 and MMP10 increased remarkably (Figure S3E).

HuR is the omnipresent member of the Hu/ELAV (human/embryonic lethal abnormal vision) RBP family and is responsible for the stabilization and (or) translation of many target mRNAs, which typically bear U-rich 3 UTRs 28-30. Importantly, previous reports demonstrated that circRNA may impair the stabilization and translation of mRNAs by competitively binding with RNA-binding protein HuR 31, 32. Considering that circDLC1 could regulate the mRNA levels of MMP1, MMP2, MMP3 and MMP10, we hypothesized that circDLC1 might act as “sponges” for HuR, leading to decreased mRNA levels of downstream targets. To test this hypothesis, we examined the RNA levels of four candidate mRNAs (MMP1, MMP2, MMP3, MMP10) after knocking down HuR with siRNA. The result showed that only the mRNA level of MMP1 was decreased (Figure 7C, D and Figure S3F). Furthermore, we examined the interaction between HuR and four candidate mRNAs (MMP1, MMP2, MMP3, MMP10). HuR could not interact with MMP2, MMP3 and MMP10 (Figure S4A). More importantly, the interaction between HuR and MMP1 mRNA in circDLC1-overexpressing cells was reduced significantly, whereas the interaction between HuR and MMP1 mRNA in circDLC1 knockdown cells was enhanced remarkably (Figure 7E-G). Taken together, these data indicated that MMP1 is the downstream target of circDLC1-HuR axis. Then we verified that protein level of MMP1 was significantly decreased in circDLC1-overexpressing cells (Figure S3G), which is consistent with effect of circDLC1 on MMP1 mRNA.

Next, we investigated the mechanism of MMP1 downregulation mediated by HuR knockdown. We found that HuR silencing decreased MMP1 mRNA levels by enhancing its degradation, because silencing HuR lowered the half-life of MMP1 mRNA significantly (Figure 7H). Moreover, we observed that the expression of MMP1 was significantly increased by circDLC1 knockdown and deletion of HuR markedly repressed the promotion (Figure 7I). In addition, we predicted the binding possibility for HuR-circDLC1 and HuR-MMP1 mRNA by two softwares, catRAPID 33 and PRIdictor 34, which indicated that HuR held a high potential to bind with circDLC1 and 3'UTR of MMP1 mRNA (Figure S4B, C). More importantly, the prediction suggested that polypeptide at 126-177 of HuR possessed high binding potential both for circDLC1 and 3' UTR of MMP1 mRNA (Figure S4D). Taken together, these observations demonstrated that circDLC1 inhibited expression of MMP1 by reducing the stability of MMP1 mRNA.

### CircDLC1 inhibits the metastasis in hepatoma cells through HuR-MMP1 axis

**Figure 8:**
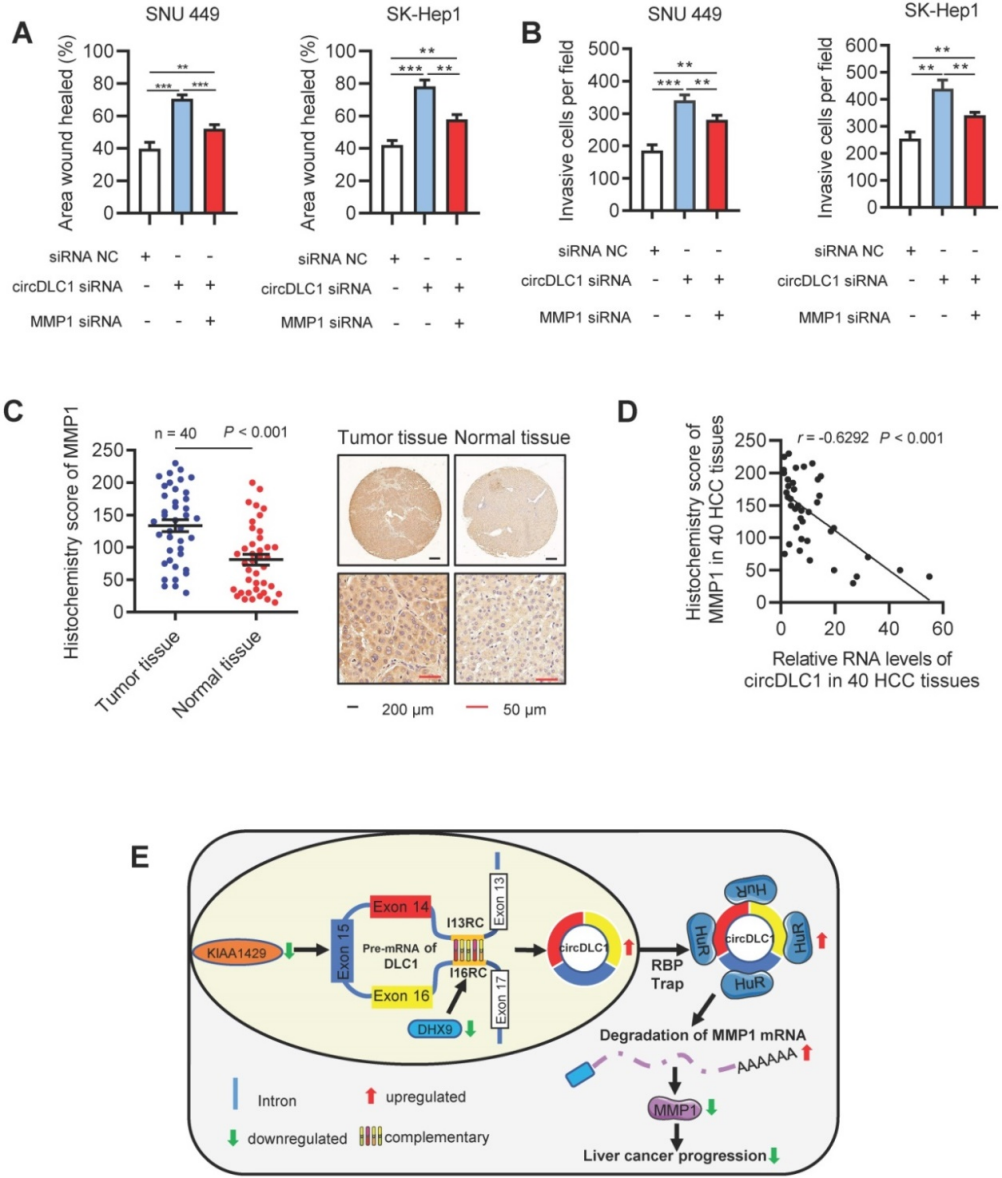
** circDLC1 inhibits the metastasis in Hepatoma cells through the HuR-MMP1 pathway. (A)** Scratch wound healing assays for indicated cells. **(B)** Transwell invasion assays for indicated cells. **(C)** IHC stains of MMP1. (Left) Histochemistry score of MMP1 in 40 HCC tissues and adjacent normal tissues. (Right) Representative samples. data are presented as mean ± SEM, Wilcoxon signed-rank test was used. **(D)** The correlation between the relative expression of circDLC1 and the IHC stains of MMP1 in 40 HCC tissues. The correlation was measured by Pearson correlation analysis. **(E)** Potential schematic pathway illustrated the role of circDLC1 in liver cancer progression. For (A) and (B), Data are presented as mean ± SD; n = 3. Student's t test was used. **P < 0.01, ***P < 0.001.

A previous study has demonstrated that MMP1 inhibited cell proliferation and invasion in HCC 35. Our data proved that MMP1 possessed the inhibition capacity of invasion and migration in hepatoma cells (Figure S5A-C). Functionally, as revealed by scratch wound healing and transwell assays, knockdown of circDLC1 could promote the motility of hepatoma cells, while the promotion could be blocked by deletion of MMP1 (Figure 8A, B and Figure S6A, B). In addition, we assessed the expression level of MMP1 in HCC samples (n = 40) by RT-qPCR and IHC. Notably, MMP1 was obviously upregulated in HCC tissues (Figure 8C and Figure S6C). We also observed that the expression of MMP1 was negatively correlated with the expression of circDLC1 in HCC tissues (Figure 8D). Taken together, these data indicated that the metastasis of HCC can be inhibited by circDLC1 via HuR-MMP1 axis.

## Discussion

Mounting evidence reveal that m6A modification plays a critical role in different human cancers 6, 35. As a primary component of the m6A methyltransferase complex, KIAA1429 has recently been demonstrated to promote HCC progression 11, 12. More importantly, growing evidence suggested that the biogenesis and function of circRNAs could be regulated by m6A modification and m6A regulated-circRNAs may provide potential therapeutic targets 17, 18. In our present work, we identified circDLC1 as a novel KIAA1429-regulated circRNA, which was clinically relevant to favorable prognosis of HCC patients. Moreover, we clarified the molecular mechanism underlying circDLC1-mediated inhibition of growth and metastasis in hepatoma cells. Our findings will facilitate the understanding of the function of m6A-regulated circRNAs and the assessment of m6A-regulated circRNAs as therapeutic targets for HCC.

Multiple studies have reported that the alternative splicing of RNA was regulated by m6A modification 6. For example, METTL3 methylated the transited adenosine in codon 273 that resulted in overexpression of R273H mutated p53 protein by enhancing the preferential pre-mRNA splicing 36. In addition, a previous study revealed that circRNAs were derived from their parental pre-mRNAs by different alternative splicing 37. In our study, we found that the expression of preDLC1 was increased in KIAA1429 knockdown cells as well as the expression of circDLC1 (data not shown), suggesting that the regulatory role of KIAA1429 in the production of circDLC1 is implicated with the processing of preDLC1. The detailed mechanism of KIAA1429-mediated alternative splicing during the processing of preDLC1 into circDLC1 will be fully illustrated in our future work. On the other hand, increasing studies suggested that the production of circRNAs can be regulated by two or more RNA-binding proteins synergistically 37. In our study, we only demonstrated that DHX9 was involved in the production of circDLC1 by binding to the flanking inverted complementary sequences, which is similar to the mechanism reported by previous studies 21, 22. However, whether other RBPs are involved in the biogenesis of circDLC1 or not requires further exploration.

Previous studies demonstrated that HuR is responsible for the stabilization and (or) translation of many target mRNAs, which typically bear U-rich 3' UTRs 27-29. For instance, HuR has been reported to promote the progression of many cancers by enhancing the expression of multiple angiogenic factors 29. Recent studies revealed that circRNAs may impair the stabilization and translation of mRNAs by competitively binding with HuR 30, 31. Consistently, in the present study, we proved that circDLC1 could reduce the interaction between HuR and MMP1 mRNA, therefore inhibit the expression of MMP1 and HCC progression. Our future work will focus on illustrating the competitive binding sites between HuR and circDLC1, as well as the binding sites between HuR and 3' UTR of MMP1 mRNA.

MMP1, a member of the zinc-dependent endopeptidase family, has been demonstrated to be closely relevant to migration and invasion in a number of cancers 38. Interestingly, recent studies revealed that MMP1 could also promote cancer cell proliferation, including HCC 34, 39. In this study, we confirmed that MMP1 is able to promote both the proliferation and metastasis of hepatoma cells. However, the detailed role of MMP1 in proliferation-related signaling pathway and the underlying mechanism remain to be investigated.

## Conclusion

In summary, our study identified circDLC1 as a novel downstream effector of KIAA1429-mediated m6A modification and confirmed the critical role of m6A modification in regulating the biogenesis of circRNAs. Furthermore, we demonstrated that circDLC1 suppresses the growth and metastasis of HCC via the HuR-MMP1 axis. Our findings shed new light on the underlying mechanisms of HCC progression and suggest that circDLC1 may serve as a prognostic biomarker and potential therapeutic target of HCC patients.

## Supplementary Material

Supplementary figures and tables.

## Abbreviations

m6A: N6-methyladenosine
circRNAs: circular RNAs
HCC: hepatocellular carcinoma
RT-qPCR: reverse transcription reaction and quantitative real-time PCR
RBPs: RNA binding proteins
HuR: ELAV-like protein 1
RIP: RNA-protein immunoprecipitation
IHC: immunohistochemistry
MMP1: matrix metallopeptidase 1
