# Supplementary material for: Circular RNA circDLC1 inhibits MMP1-mediated liver cancer progression via interaction with HuR
Source: Theranostics. 2021 Jan 1;11(3):1396–411. doi: 10.7150/thno.53227 (PMC7738888; doi:10.7150/thno.53227)
Supplement: Supplementary file 1 — Supplementary figures and tables. [file thnov11p1396s1.zip › Supplementary materials.pdf]

Supplementary materials and methods

RNA sequencing

For circRNAs-analysis dataset, differentially expressed circRNAs were analyzed from the whole transcriptome sequencing of shCtrl cells and shKIAA1429 cells [1]. For the mRNA sequencing, total RNA was extracted from lv-vector cells and LV-circDLC1 cells using Trizol Reagent (Invitrogen). mRNA Sequencing libraries used the NEB Next Ultra RNA Library Prep Kit (Illumina) as previously described by CapitalBio Technology (Beijing, China) [2].

RNase R treatment

For RNase R treatment, total RNA was extracted by using Trizol reagent (Invitrogen, CA, USA), 3 μg total RNA were incubated with 10U RNase R (20U/μl, Epicentre, Madison, WI, USA) in a 10 μl volume at 37 °C for 30min, followed by 75 °C for 10min to deactivate the RNase R. The treated RNAs were analyzed by RT-qPCR.

In vitro cell-behaviour assays

For CCK-8 assays, 3000 cells /100 μl DMEM were equally seeded in 96-wells plates and incubated for four consecutive days. Cell viability was measured by using Cell Counting Kit-8 (CCK-8) (Beyotime Biotechnology, Shanghai, China), according to the manufacturer's instructions. Cell cycle was conducted with Cell Cycle Kit (4A Biotech, Beijing, China), according to the manufacturer's protocols. CytoFLEX Research Flow Cytometer (Beckman Coulter, CA, USA) was used for analyzing data. 1×10^6 cells/well

was proper density in 6-well plates for the wound-healing assays. Confluent cell monolayers were wounded by scraping with a 100 μL plastic pipette tip. After 24 or 72h, the proportion of wound-healing changed area was calculated by Image J (National Institutes of Health, USA). For migration and invasion assays, Transwell chambers (Costar, Corning, NY) were put into 24-well plates which contain DMEM with 10%FBS. Especially for invasion assays, chambers were precoated with Matrigel. 20000 cells for migration/chamber and 50000 cells for invasion/chamber are the proper cell density for assays. The number of cells in each chamber was counted by using Image J (National Institutes of Health, USA).

Construction of stable cell lines with overexpression and knockdown of circDLC1

For knockdown of circDLC1, the same cDNA oligonucleotides with circDLC1 siRNA was synthesized with annealing, double-strand oligonucleotides were inserted into shRNA expression plasmid pWSLV-sh08 (GeneCopoeia, CA, USA), named pWSLV-sh08- circ_0135718. The circDLC1-overexpressing plasmid synthesized by Geneseed (Guangzhou, China) named pLCDH-circDL1. All plasmids were transfected by using HEK-293T cells, Viruses were produced in HEK-293T cells as previously described [3].

Northern blot analysis

The circDLC1 junction probe and 18s probe were synthesized and labeled by digoxin(Supplementary table 6). Northern blot analysis was conducted with a Northern

blot kit (Ambion), according to the manufacturer's protocols. In brief, 15 μg RNA was electrophoresed in a 1% agarose–formaldehyde gel under denaturing conditions overnight at 25V, and then transferred onto a Hybond-N+nylon membrane (Amersham). Digoxigenin-labelled DNA probes were hybridized with Hybond-N+nylon membrane and subsequently treated with anti-DIG antibody staining. The intensity of signals was scanned by X-ray films with chemiluminescence substrate CSPD (Roche).

Subcellular RNA fractionation

Cytoplasmic and nuclear RNA fractions were performed with the PARIS^{TM} Kit (Invitrogen, CA, USA), according to the instructions, subsequently analyzed by RT-qPCR. β-actin mRNA was used as the cytoplasmic endogenous control. U3 small nuclear RNA was used as the nuclear endogenous control.

Subcellular protein fractionation

Cytoplasmic and nuclear protein fractions were conducted with the NE-PER^{TM} Nuclear and Cytoplasmic Extraction Reagents (Thermo Fisher Scientific, CA, USA), according to the manufacturer's protocols. Histone H3 was used as the nuclear endogenous control. β-actin was used as the cytoplasmic endogenous control.

Immunohistochemistry (IHC) assay

For the Immunohistochemistry (IHC) assay, HCC specimens tissue slides were deparaffinized and rehydrated using xylene and ethanol followed by antigen retrieval

with sodium citrate buffer. All sections were treated with the first antibody against KIAA1429 (Novus Biologicals, CO, USA), DHX9 (Abcam, Cambridge, USA), MMP1(Proteintech, WuHan, China) overnight at 4°C. Finally, IHC staining was performed with horseradish peroxidase (HRP) conjugates using DAB detection. The histochemistry score was assessed as our previous work [4].

Reverse transcription reaction and quantitative real-time PCR

Total RNA was extracted by using Trizol reagent (Invitrogen, CA, USA). First-strand cDNA was generated using the PrimeScript™ Reverse Transcriptase kit (Takara, Shiga, Japan) with random primers. Real-time quantitative PCR was performed with a ChamQ™ SYBR@ qPCR Master Mix (Vazyme Biotech, Nanjing, China). All reactions were performed in triplicate. β-actin or U6 were employed as endogenous control. The relative expression was calculated using the comparative Ct ($2^{-\Delta\Delta}$CT) method. The primer sequences are presented in (Supplementary table 5).

Immunofluorescence (IF)

HCC cells were fixed with 4% formaldehyde for 20 min and then blocked with 5% normal goat serum for 1h. Immunostaining was treated with antibody specific HuR (Abcam, Cambridge, USA, 1:150 dilution) overnight at 4°C. Then, cells were treated with Cy5-conjugated goat anti-mouse IgG (1:1000 dilution). Images were acquired by the A1RþMP Confocal Laser Microscope System (Nikon).

Cell transfection

The siRNAs were transfected by using Genmute^{TM} Reagent (SignaGen Laboratories, Maryland, USA) according to the manufacturer's protocol. The cells were harvested at 48h after transfection. GenJetTM Plus reagent (SignaGen Laboratories, Maryland, USA) was used for cell transfection with plasmids. The cells were harvested at 72h after transfection.

Actinomycin D assay

$5x10^5$ cells were equally seeded in 6 wells plate. 24 hours later, the cells were respectively exposed to actinomycin D (1μg/ml) for 0h, 4h, 8h, 12h, 24h. The relative RNA levels of circDLC1 and mDLC1 were analyzed by RT-qPCR.

Western blot analysis

Total protein was extracted by RIPA Lysis Buffer (Beyotime Biotechnology, Shanghai, China) and protease inhibitor cocktail (Beyotime Biotechnology, Shanghai, China). The BCA Protein Assay Kit (Beyotime Biotechnology, Shanghai, China) was used for detecting protein concentration. Proteins were electrophoresed in sodium dodecyl sulfate-polyacrylamide gel electrophoresis (SDS-PAGE) and transferred onto PVDF membranes (Millipore, Massachusetts, USA). The membranes were incubated with primary antibodies and horseradish peroxidase (HRP)-conjugated secondary antibodies successively. The enhanced chemiluminescent (ECL) chromogenic substrate (Millipore, Massachusetts, USA) was used for visualizing immunoreactivity. The

intensity of signals was detected by ChemiDoc MP Imager System (Bio-Rad, California, USA) and analyzed by Image Lab 5.2 software (Bio-Rad, California, USA).

Vector construction

The genomic region for circDLC1 with its wild-type flanking introns (a 3754-nt region of the DLC1 gene, spanning from intron 13 to intron 16) was amplified from Huh7 genomic DNAs. Wild type pEZ-hsa_circ_0135718-Lv201 (GeneCopoeia, CA, USA) plasmid was cloned by inserting the 3754-nt region of the DLC1 gene. DNA sequences with I13RC and (or) I16RC depletion were synthesized by PEPTBIO (PEPTBIO, Wuhan, China) and cloned into pEZ- Lv201 vector as mentioned above.

References

1. Lan T, Li H, Zhang D, Xu L, Liu H, Hao X, et al. KIAA1429 contributes to liver cancer progression through N6-methyladenosine-dependent post-transcriptional modification of GATA3. Mol Cancer. 2019; 18: 186.
2. Chen R-X, Chen X, Xia L-P, Zhang J-X, Pan Z-Z, Ma X-D, et al. N6-methyladenosine modification of circNSUN2 facilitates cytoplasmic export and stabilizes HMGA2 to promote colorectal liver metastasis. Nature Communications. 2019; 10.
3. Olmeda D, Cerezo-Wallis D, Riveiro-Falkenbach E, Pennacchi PC, Contreras-Alcalde M, Ibarz N, et al. Whole-body imaging of lymphovascular niches identifies pre-metastatic roles of midkine. Nature. 2017; 546: 676-80.
4. Li H, Lan T, Xu L, Liu H, Wang J, Li J, et al. NCSTN promotes hepatocellular carcinoma cell growth and metastasis via β-catenin activation in a Notch1/AKT dependent manner. Journal of Experimental & Clinical Cancer Research. 2020; 39.

Supplementary figures

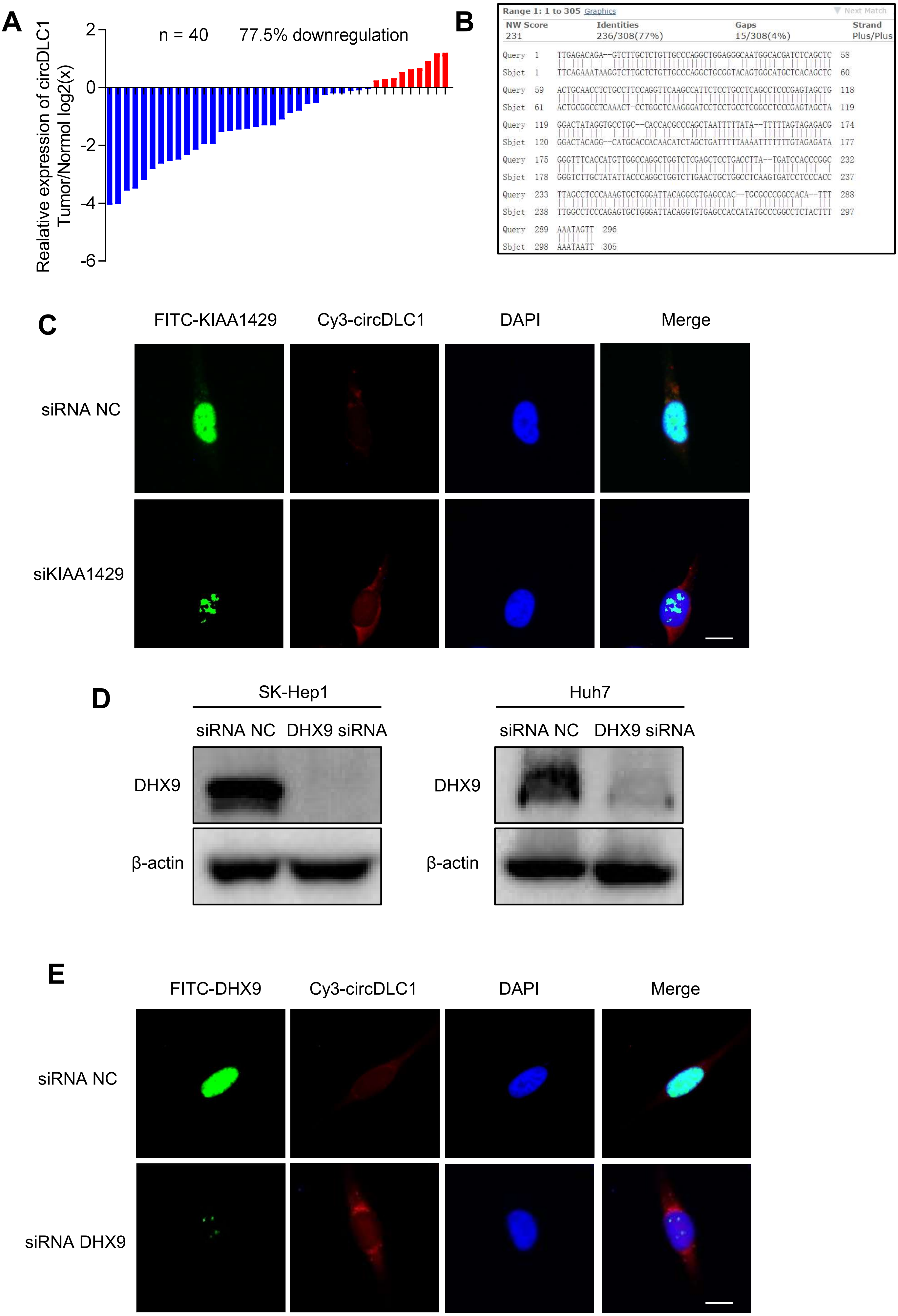
A
B
C
D
E
2
0
-2
-4
-6
n = 40
77.5% downregulation
Realative expression of circDLC1
Tumor/Normol log2(x)
Range 1: 1 to 305 Graphics
Next Match
NW Score 231
Identities 236/308(77%)
Gaps 15/308(4%)
Strand Plus/Plus
Query 1 TTGAGACAGA--GTCTTGCTCTGTTGCCCAGGCTGGAGGGCAATGGCACGATCTCAGCTC 58
Sbjct 1 TTCAGAAATAAGGTCTTGCTCTGTTGCCCAGGCTGCGGTACAGTGGCATGCTCACAGCTC 60
Query 59 ACTGCAACCTCTGCCTTCCAGGTTCAAGCCATTCTCCTGCCTCAGCCTCCCGAGTAGCTG 118
Sbjct 61 ACTGCGGCCTCAAACT-CCTGGCTCAAGGGATCCTCCTGCCTCGGCCTCCCGAGTAGCTA 119
Query 119 GGACTATAGGTGCCTGC--CACCACGCCCAGCTAATTTTTATA--TTTTTAGTAGAGACG 174
Sbjct 120 GGACTACAGG--CATGCACCACAACATCTAGCTGATTTTTAAAATTTTTTTGTAGAGATA 177
Query 175 GGGTTTCACCATGTTGGCCAGGCTGGTCTCGAGCTCCTGACCTTA--TGATCCACCCGGC 232
Sbjct 178 GGGTCTTGCTATATTACCCAGGCTGGTCTTGAACTGCTGGCCTCAAGTGATCCTCCCACC 237
Query 233 TTAGCCTCCCAAAGTGCTGGGATTACAGGCGTGAGCCAC—TGCGCCCGGCCACA--TTT 288
Sbjct 238 TTGGCCTCCCAGAGTGCTGGGATTACAGGTGTGAGCCACCATATGCCCGGCCTCTACTTT 297
Query 289 AAATAGTT 296
Sbjct 298 AAATAATT 305
FITC-KIAA1429
Cy3-circDLC1
DAPI
Merge
siRNA NC
siKIAA1429
SK-Hep1
Huh7
siRNA NC
DHX9 siRNA
DHX9
β-actin
FITC-DHX9
siRNA DHX9

Figure S1

(A) Expression levels of circDLC1 in 40 HCC and their adjacent normal tissues by RT-qPCR. **(B)** The sequence of intron 13 of DLC1 gene was aligned to that of intron 16 of the DLC1 gene using BLAST (https://blast.ncbi.nlm.nih.gov/Blast.cgi). We found highly reverse complementary sequences (77% identity over 230nt) and termed them I13RC (reverse complementary sequences in intron 13) and I16RC (reverse complementary sequences in intron 16), respectively. Query, intron 13. Sbjct, intron16. **(C)** Expression of circDLC1 was examined by FISH and IF with KIAA1429 knockdown cells. Nuclei were stained with DAPI. Scale bar, 10 μm. **(D)** Western blot showed the protein level of DHX9 was successfully knocked down using siRNAs. **(E)** Expression of circDLC1 was examined by FISH and IF with DHX9 knockdown cells. Nuclei were stained with DAPI. Scale bar, 10 μm.

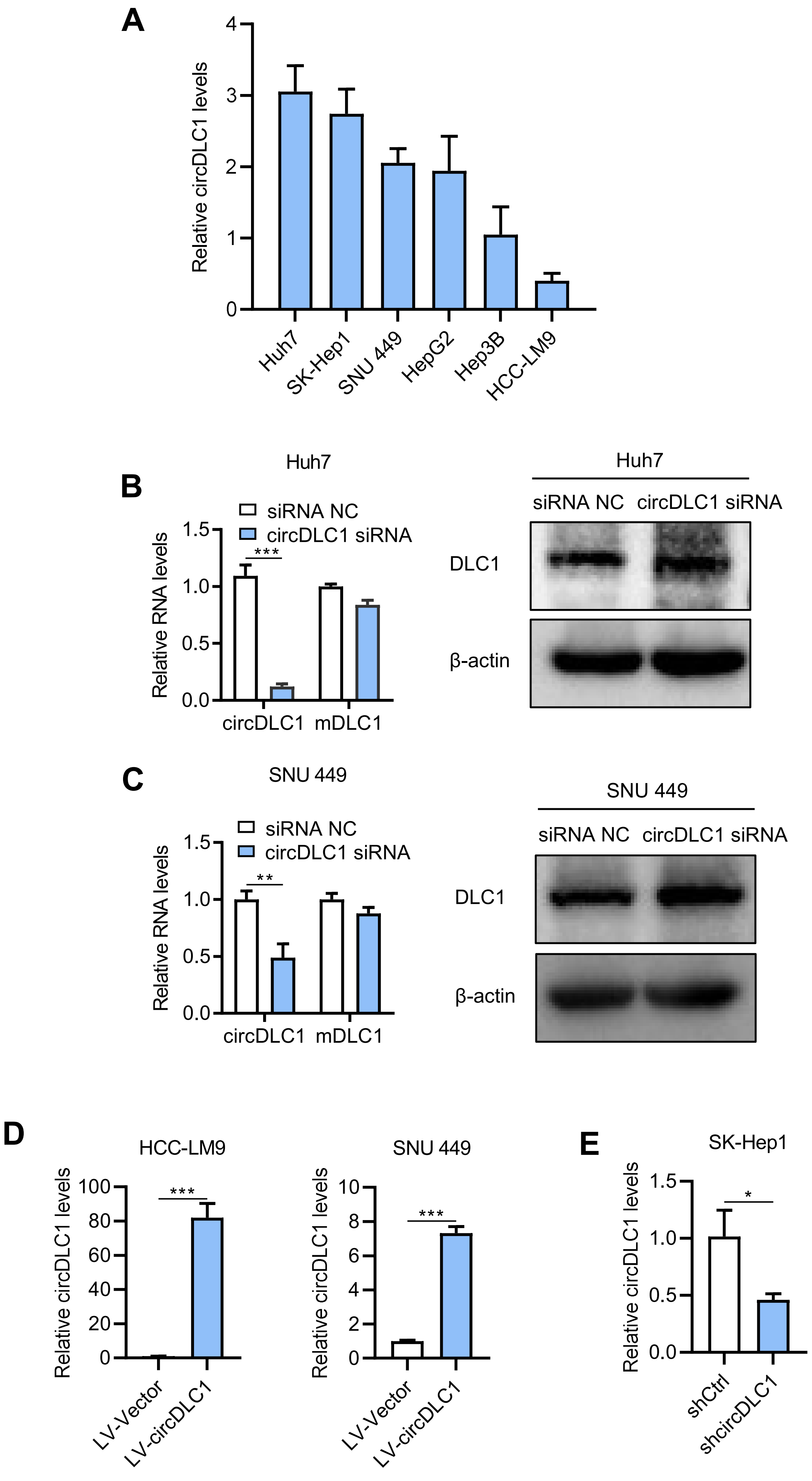
A
Relative circDLC1 levels
0
1
2
3
4
Huh7
SK-Hep1
SNU 449
HepG2
Hep3B
HCC-LM9
B
Huh7
siRNA NC
circDLC1 siRNA
***
Relative RNA levels
0.0
0.5
1.0
1.5
circDLC1
mDLC1
Huh7
siRNA NC
circDLC1 siRNA
DLC1
β-actin
C
SNU 449
siRNA NC
circDLC1 siRNA
**
Relative RNA levels
0.0
0.5
1.0
1.5
circDLC1
mDLC1
SNU 449
siRNA NC
circDLC1 siRNA
DLC1
β-actin
D
HCC-LM9
Relative circDLC1 levels
0
20
40
60
80
100
***
LV-Vector
LV-circDLC1
SNU 449
Relative circDLC1 levels
0
2
4
6
8
10
***
LV-Vector
LV-circDLC1
E
SK-Hep1
Relative circDLC1 levels
0.0
0.5
1.0
1.5
*
shCtrl
shcircDLC1

Figure S2

(A) The expression of circDLC1 in a variety of human hepatoma cell lines by RT-qPCR. **(B)** RT-qPCR and western blot showed the expression of circDLC1 and DLC1 after silencing circDLC1 in Huh7 cells. **(C)** RT-qPCR and western blot showed the expression of circDLC1 and DLC1 after silencing circDLC1 in SNU 4449 cells. **(D)** RT-qPCR showed the expression of circDLC1 after overexpressing circDLC1 in HCC-LM9 and SNU 449 cells. **(E)** RT-qPCR showed the expression of circDLC1 SK-Hep1 with stable knockdown circDLC1. Data are presented as mean ± SD; n = 3. Student's t test was used. *P<0.05, **P < 0.01, ***P < 0.001.

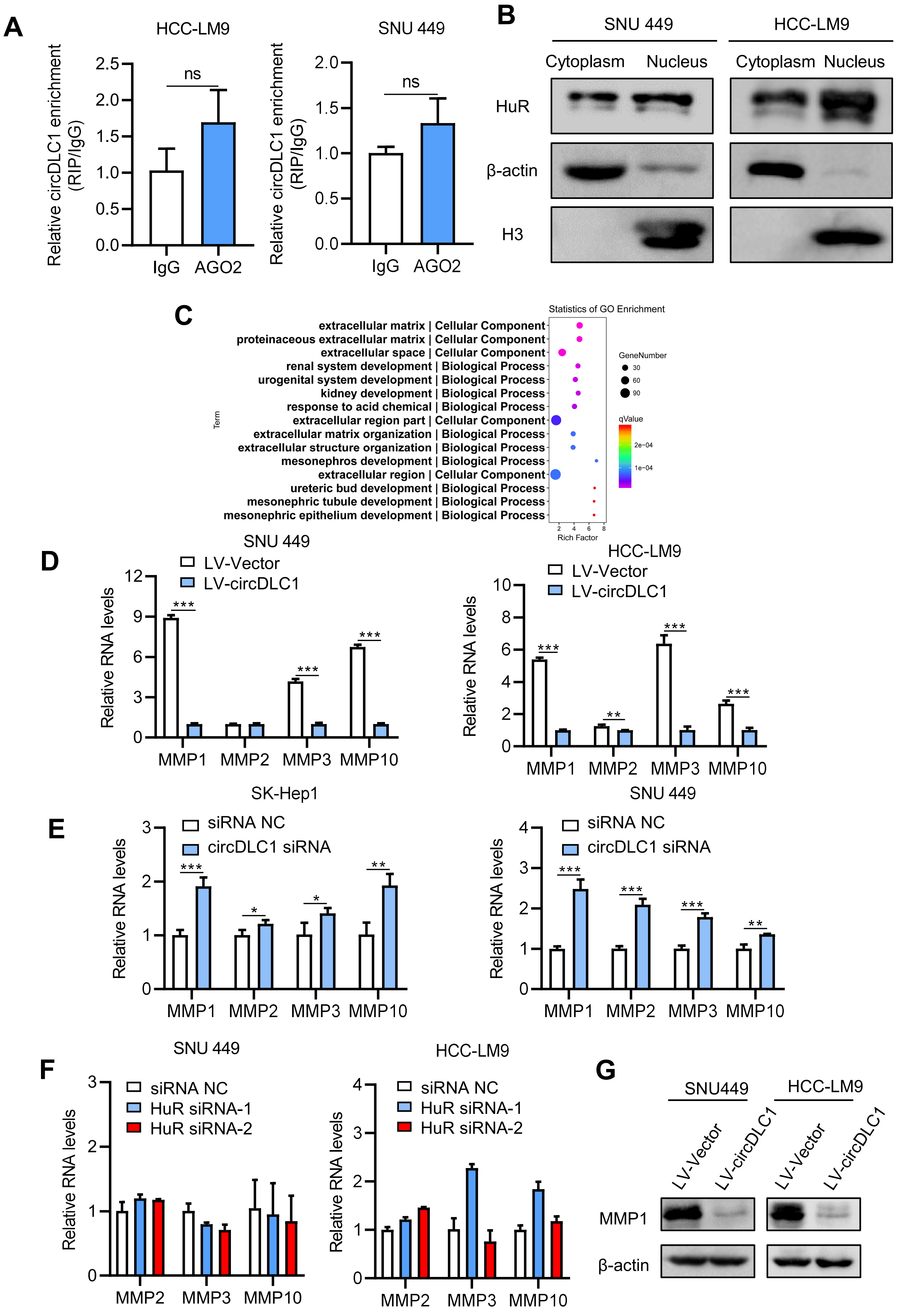
A
HCC-LM9
SNU 449
Relative circDLC1 enrichment (RIP/IgG)
ns
IgG
AGO2
B
SNU 449
HCC-LM9
Cytoplasm
Nucleus
HuR
β-actin
H3
C
Statistics of GO Enrichment
extracellular matrix | Cellular Component
proteinaceous extracellular matrix | Cellular Component
extracellular space | Cellular Component
renal system development | Biological Process
urogenital system development | Biological Process
kidney development | Biological Process
response to acid chemical | Biological Process
extracellular region part | Cellular Component
extracellular matrix organization | Biological Process
extracellular structure organization | Biological Process
mesonephros development | Biological Process
extracellular region | Cellular Component
ureteric bud development | Biological Process
mesonephric tubule development | Biological Process
mesonephric epithelium development | Biological Process
Term
GeneNumber
qValue
Rich Factor
D
SNU 449
HCC-LM9
LV-Vector
LV-circDLC1
Relative RNA levels
MMP1
MMP2
MMP3
MMP10
E
SK-Hep1
SNU 449
siRNA NC
circDLC1 siRNA
F
SNU 449
HCC-LM9
siRNA NC
HuR siRNA-1
HuR siRNA-2
G
SNU449
HCC-LM9
LV-Vector
LV-circDLC1
MMP1
β-actin

Figure S3

(A) RIP experiments were performed using an antibody against AGO2 on extracts from HCC cells. **(B)** The subcellular location of HuR measured by western blot. **(C)** Gene Ontology (GO) analysis showed the enriched pathway of circDLC1 target genes. **(D)** The mRNA levels of MMP1, MMP2, MMP3 and MMP10 in circDLC1-overexpressing cells were determined by RT-qPCR. **(E)** The mRNA levels of MMP1, MMP2, MMP3 and MMP10 in circDLC1-knockdown cells were determined by RT-qPCR. **(F)** RT-qPCR showed the expression of MMP2, MMP3 and MMP10 after silencing HuR. **(G)** The expression of MMP1 was detected by western blot with stable circDLC1-overexpressing cells. Data are presented as mean ± SD; n = 3. Student's t test was used. $^{**}P < 0.01$, $^{***}P < 0.001$.

A

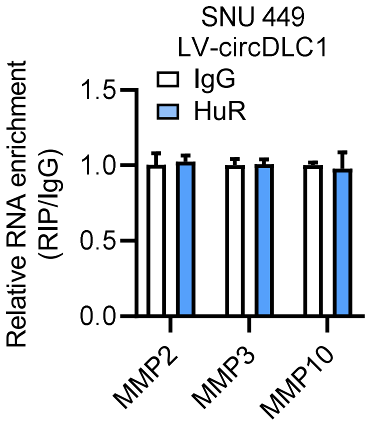

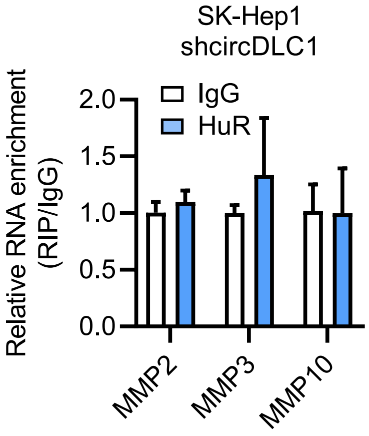

B HuR and circDLC1 binding potential

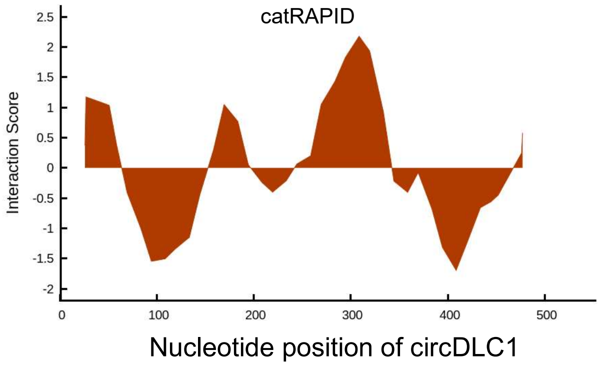

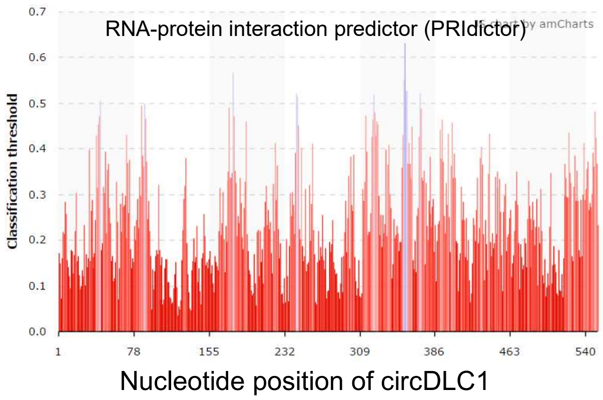

C HuR and MMP1 3'UTR binding potential

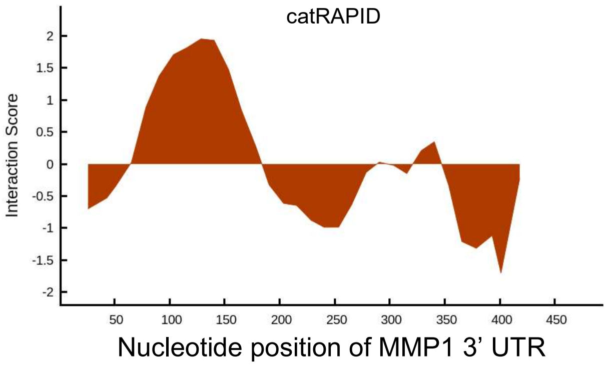

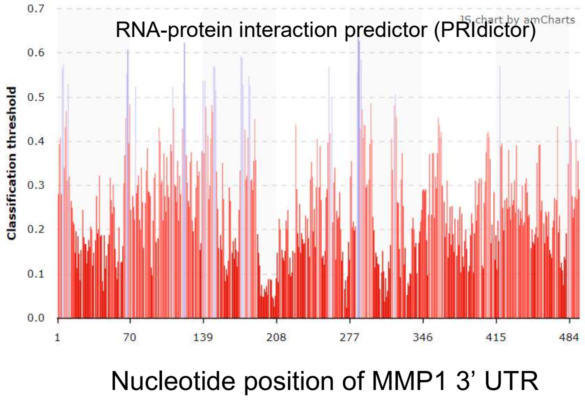

D

Interaction region between HuR and circDLC1

#	Protein region	RNA region	Interaction Propensity	Discriminative Power	Normalized Score
1	251-302	301-352	6.40	22	2.22
2	176-227	301-352	5.81	22	2.03
3	176-227	301-352	5.81	22	2.03
4	126-177	202-253	5.52	22	1.93
5	126-177	202-253	5.52	22	1.93
6	251-302	276-327	4.59	20	1.63
7	251-302	277-328	4.55	20	1.62
8	126-177	27-78	4.53	20	1.61
9	126-177	27-78	4.53	20	1.61
10	26-77	301-352	4.51	20	1.60
11	26-77	301-352	4.51	20	1.60
12	76-127	301-352	4.47	20	1.59
13	76-127	301-352	4.47	20	1.59
14	151-202	301-352	4.23	20	1.51
15	151-202	301-352	4.23	20	1.51
16	251-302	302-353	4.18	20	1.50
17	176-227	276-327	4.18	20	1.50
18	176-227	276-327	4.18	20	1.50
19	176-227	277-328	4.13	20	1.48
20	176-227	277-328	4.13	20	1.48

Interaction region between HuR and MMP1 3'UTR

#	Protein region	RNA region	Interaction Propensity	Discriminative Power	Normalized Score
1	126-177	118-169	6.26	22	2.42
2	126-177	118-169	6.26	22	2.42
3	126-177	143-194	4.67	20	2.04
4	126-177	143-194	4.67	20	2.04
5	126-177	126-177	4.23	20	1.94
6	126-177	126-177	4.23	20	1.94
7	126-177	318-369	3.93	20	1.86
8	126-177	318-369	3.93	20	1.86
9	126-177	101-152	3.23	20	1.70
10	126-177	101-152	3.23	20	1.70
11	126-177	151-202	2.66	17	1.56
12	126-177	151-202	2.66	17	1.56
13	226-277	318-369	2.45	17	1.51
14	226-277	318-369	2.45	17	1.51
15	126-177	301-352	2.35	17	1.49
16	126-177	301-352	2.35	17	1.49
17	126-177	93-144	2.33	17	1.48
18	126-177	93-144	2.33	17	1.48
19	126-177	168-219	2.25	17	1.46
20	126-177	168-219	2.25	17	1.46

Figure S4

(A) RIP experiments were performed using an antibody against HuR with extracts from hepatoma cells. **(B)** The binding potential between HuR and circDLC1 was predicted online. **(C)** The binding potential between HuR and 3'UTR of MMP1 mRNA was predicted online. **(D)** The binding sites of HuR-circDLC1 and HuR-3'UTR of MMP1 mRNA binding were predicted online. The binding sites of HuR are indicated by red bars.

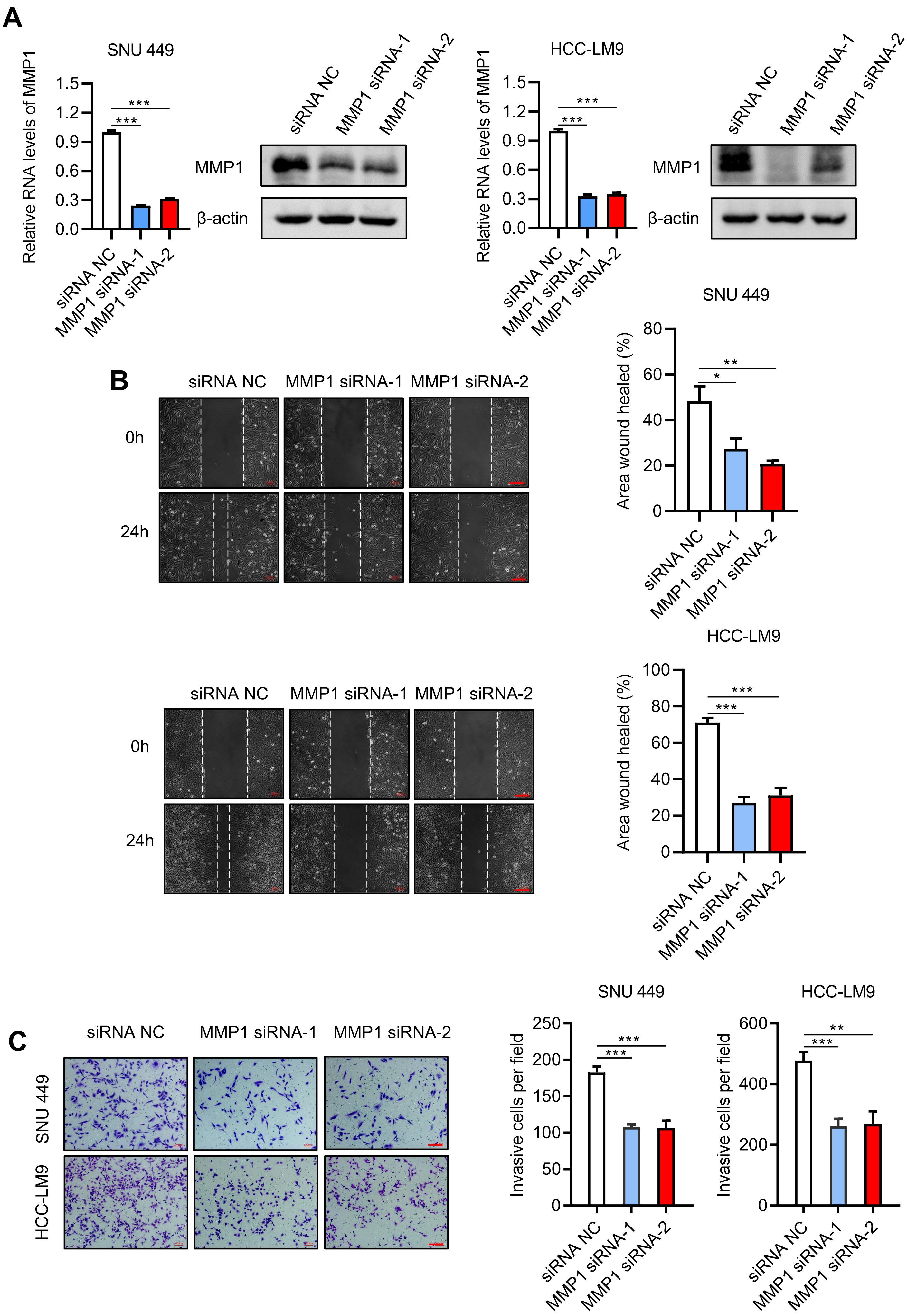
A
SNU 449
Relative RNA levels of MMP1
1.5
1.2
0.9
0.6
0.3
0.0
***
***
siRNA NC
MMP1 siRNA-1
MMP1 siRNA-2
MMP1
β-actin
HCC-LM9
B
0h
24h
Area wound healed (%)
80
60
40
20
0
**
*
100
C
SNU 449
HCC-LM9
Invasive cells per field
250
200
150
100
50
600
400
200

Figure S5

(A) Western blot and RT-qPCR showed the expression of MMP1 after silencing MMP1 in HCC cells. **(B)** Scratch wound healing assays for indicated cells. **(C)** Transwell assays for indicated cells. Scale bars = 200 μm. Data are presented as mean ± SD; n = 3. Student's t test was used. *P<0.05, **P < 0.01, ***P < 0.001.

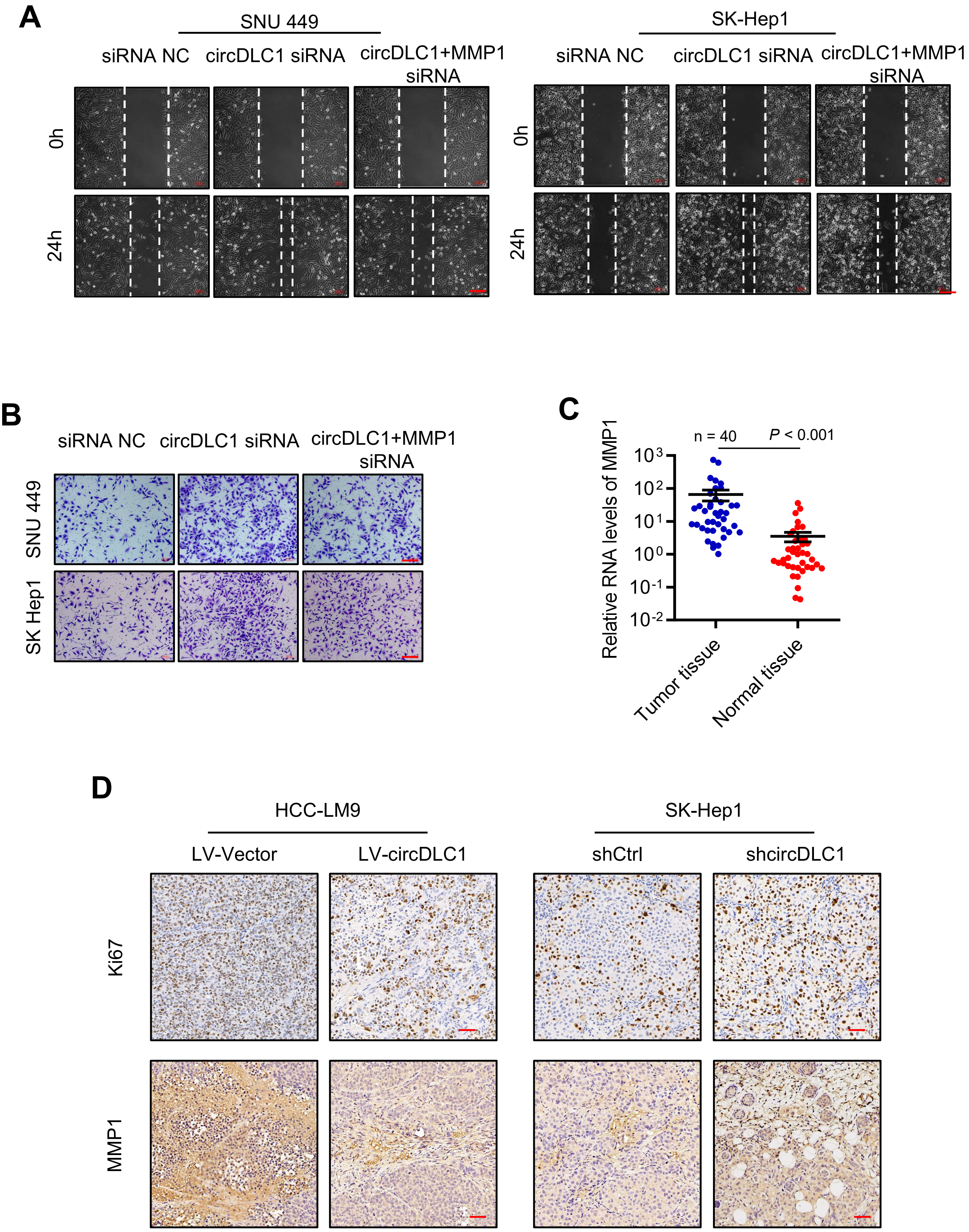
A
SNU 449
siRNA NC
circDLC1 siRNA
circDLC1+MMP1 siRNA
SK-Hep1
siRNA NC
circDLC1 siRNA
circDLC1+MMP1 siRNA
0h
24h
0h
24h
B
siRNA NC
circDLC1 siRNA
circDLC1+MMP1 siRNA
SNU 449
SK Hep1
C
n = 40
P < 0.001
Relative RNA levels of MMP1
10^3
10^2
10^1
10^0
10^-1
10^-2
Tumor tissue
Normal tissue
D
HCC-LM9
SK-Hep1
LV-Vector
LV-circDLC1
shCtrl
shcircDLC1
Ki67
MMP1

Figure S6

(A) Scratch wound healing assays for indicated cells. **(B)** Transwell assays for indicated cells. **(C)** The relative mRNA levels of MMP1 expression in 40 pairs of HCC tissues and adjacent normal tissues by using RT-qPCR. Scale bars = 200 μm. The data are presented as mean ± SEM, Wilcoxon signed-rank test was used. **(D)** Representative images of corresponding Ki-67 and MMP1 staining of subcutaneously HCC tumor, Scale bars = 50 μm.

Table S1. Clinical baseline characteristics of 110 patients with HCC according to CircDLC1 expression level.

Variable	circDLC1 Low	circDLC1 High	p value
All patients	47	63	
Age, years, >50: ≤50	25:22	27:36	0.542
Gender, male/female	37:10	55:8	0.229
HBSAg, positive/negative	39:8	57:6	0.2431
Liver cirrhosis, present/absent	30:17	36:27	0.4788
AFP, ug/L, >20: ≤20	32:15	37:26	0.3155
Tumor size, cm, >5: ≤5	24:23	36:27	0.0999
No. tumor, multiple: solitary	14:33	12:51	0.1897
Microvascular invasion, present/absent	25:22	18:45	0.0089*
Macrovascular invasion, present/absent	3:44	3:60	0.999
Tumor differentiation, poor/well, moderate	27:20	22:41	0.0187*
TNM stage, II+III: I	30:17	24:39	0.0076*
BCLC stage, B+C: A	21:26	12:51	0.0037*

Chi-square test was used to test the association between two categorical variables. AFP, alpha-fetoprotein; BCLC Barcelona Clinic Liver Cancer; HBsAg, hepatitis B surface antigen.

* Statistically significant.

Table S2. Prognostic factors for overall survival and recurrence-free survival by the univariate Cox proportional hazards regression model.

Variables	Overall survival			Recurrence-free survival		
	**HR**	**95% CI**	***P***	**HR**	**95% CI**	***P***
Age, ≤50/>50	0.825	0.508-1.339	0.436	0.706	0.429-1.161	0.17
Gender, F/M	0.911	0.490-1.692	0.768	0.863	0.462-1.614	0.645
HBsAg, negative/positive	0.879	0.437-1.767	0.717	0.763	0.41-1.253	0.935
AFP, <400/≥400	0.624	0.389-1.001	0.051	0.547	0.333-0.869	0.017
Ascites, -/+	0.524	0.286-0.959	0.036	0.653	0.322-1.327	0.239
Tumor size, ≤5/>5	0.672	0.421-1.073	0.095	0.853	0.525-1.386	0.521
No. tumor, solitary/ multiple	0.699	0.411-1.18	0.108	0.636	0.369-1.096	0.103
Differentiation (Poor/Well-Moderate)	0.837	0.534-1.335	0.454	0.789	0.481-1.292	0.346
TNM stage, I/II -III	0.562	0.354-0.893	0.015	0.412	0.25-0.678	<0.001
BCLC stage, A/ B+C	0.64	0.393-1.044	0.074	0.538	0.325-0.890	0.016
Macrovascular invasion, absent/present	0.390	0.156-0.970	0.043	0.281	0.109-0.721	0.008
Microvascular invasion, absent/present	0.423	0.226-0.673	<0.001	0.332	0.203-0.546	<0.001
Cirrhosis, -/+	1.088	0.681-1.739	0.725	1.212	0.739-1.987	0.445
circDLC1 (low/high)	2.669	1.676-4.251	<0.001	2.433	1.489-3.976	<0.001

TNM, tumor-node-metastasis; HR, hazard ratio; CI, confidence interval; F, female; M, male.

Table S3. Independent prognostic factors for overall survival and recurrence-free survival by the multivariate Cox proportional hazards regression model.

Variables	Overall survival			Recurrence-free survival		
	**HR**	**95% CI**	***P***	**HR**	**95% CI**	***P***
AFP, ≥400/<400				1.587	1.079-2.334	0.019
Ascites, -/+	0.508	0.273-0.948	0.033			
TNM stage, I/II -III	0.641	0.519-2.902	0.641	0.814	0.302-2.196	0.685
BCLC stage, B+C/A				1.093	0.556-2.148	0.797
Macrovascular invasion	0.543	0.196-1.127	0.216	0.6	0.212-1.694	0.335
Microvascular invasion	0.469	1.482-3.208	0.09	0.509	0.209-1.24	0.137
circDLC1, low/high	2.577	1.591-4.173	<0.001	2.078	1.238-3.488	0.006

HR, hazard ratio; CI, confidence interval.

Table S4. The antibodies used in this study

Antibody	Supplier	Catalogue number
KIAA1429	Novus	NBP1-85118
KIAA1429	Proteintech	25712-1-AP
DHX9	Abcam	ab26271
HuR	Abcam	ab136542
HuR	Abclon	A19622
MMP1	Proteintech	10371-2-AP
DLC1	Proteintech	10371-2-AP
H3	Affinity	AF6359
β-actin	Santa Cruz	sc-69879
Ki-67	Servicebio	GB13030-2
AGO2	Merck Millipore	MABE253

Table S5. Sequence of primers used for PCR in this study

Primer	Sequences
CircDLC1 forward	5'- TCGAGACTACGTTGTTTTAAGGGTA -3'
CircDLC1 reverse	5'- AGGGGCTTCAGCTCTTGTTC -3'
KIAA1429 forward	5'-CTTGGCAAGTGGCTTGAACC-3'
KIAA1429 reverse	5'-ACGTAAGGCAGTGGTAAGGC-3'
HuR forward	5-GGGTGACATCGGGAGAACGAAT-3’
HuR reverse	5’-TGTCCTGCTACTTTATCCCGAA-3’
U6 forward	5’-GCTTCGGCAGCACATATACTAAAAT-3’
U6 reverse	5’- CGCTTCACGAATTTGCGTGTCAT -3’
DHX9 forward	5’-TGCTGGGCTTCATGGAAACT -3’
DHX9 reverse	5’-TGCAATAAAGCTCCTGTTGTGA-3’
U3 forward	5'- TTCTCTGAGCGTGTAGAGCACCGA -3'
U3 reverse	5'- GATCATCAATGGCTGACGGCAGTT -3'
mDLC1 forward	5'-GCTGCAGAGGGTGTTTTTGT-3'
mDLC1 reverse	5'-GAAAGAAGTCCGTCCCCGTT-3'
preDLC1 forward	5'-GTGGACTCACCTCCTTTCTGT-3'
preDLC1 reverse	5'-CGAGTGCTTCCAGAGTGAGG-3'
ciRS-7 forward	5'-TCAACTGGCTCAATATCCATGTC-3'
ciRS-7 reverse	5'-ACCTTGACACAGGTGCCAT-3'
I13RC forward	5'-ATGGCACGATCTCAGCTCAC-3'
I13RC reverse	5'-GCTAAGCCGGGTGGATCATA-3'
I16RC forward	5'-ACTTGAGGCCAGCAGTTCAA-3'
I16RC reverse	5'-GCCTCAAACTCCTGGCTCAA-3'
MMP1 forward	5'-TGTGGTGTCTCACAGCTTCC-3'
MMP1 reverse	5'-CAACTTGCCTCCCATCATTCT-3'
MMP2 forward	5'- ACGGACAAAGAGTTGGCAGT-3'
MMP2 reverse	5'- GGGGCAGTCCAAAGAACTTCT-3'
MMP3 forward	5'- ATTGGATGGAGCTGCAAGGG-3'
MMP3 reverse	5'- CCACTGTCCTTTCTCCTAACAAAC-3'
MMP10 forward	5'- GCCAGTCTGCTCTGCCTATC-3'
MMP10 reverse	5'- CTGTTTCACATCCTTTTCGAGGT-3'
β-actin forward	5'- GGGAAATCGTGCGTGACATTAAG-3'
β-actin reverse	5'- TGTGTTGGCGTACAGGTCTTTG-3'
18S rRNA forward	5’-ACACGGACAGGATTGACAGA-3’
18S rRNA reverse	5’-GGACATCTAAGGGCATCACA-3’

Table S6. Sequence of probe used for PCR in this study

Probe name	Sequences (5'-3')	supplier
circDLC1 Pulldown Probe	CTTTGCATTACCCTTAAAACAACGTAGTCTCG-biotin	Sangon Biotech (Shanghai) Co., Ltd.
Control Pulldown Probe	GAAACGTAATGGGAATTTTGTTGCATCAGAGC-biotin	Sangon Biotech (Shanghai) Co., Ltd.
circDLC1 FISH Probe	CTTTGCATTACCCTTAAAACAACGTAGTCTCG-Cy3	Sangon Biotech (Shanghai) Co., Ltd.
circDLC1 Northern Probe	digoxin-TGGGATGTAGACCTGTTGGATTCAAAAGTGATCGAAATTCTGGACAGCCAAACTGAAATTTACCAGTATGTCCAAAACAGTATGGCACCTCATCCTGCTCGAGACTACGTTGTTTTAAGGGTAATGCAAAGAAAACAAAGTTTGGGCAAACCAGATCAGAAAGATTTGAATGAAAACCTAGCTGCCACTCAAGGGCTGGCCCATATGATCGCCGAGTGCAAGAAGCTTTTCCAGGTTCCCGAGGAAATGAGCCGATGTCGTAATTCCTATACCGAACAAGAGCTGAAGCCCCTCACTCTGGAAGCACTCGGGCACCTGGGTAATGATGACTCAGCTGACTACCAACACTTCCTCCAG-digoxin	Sangon Biotech (Shanghai) Co., Ltd.
18S Northern Probe	digoxin-GTTCCTTTGGTCGCTCGCTCCTCTCCCACTTGGATAACTGTGGTAATTCTAGAGCTAATACATGCCGACGGGCGCTGACCCCCTTCGCGGGGGGGATGCGTGCATTTATCAGATCAAAACCAACCCGGTCAGCCCCTCTCCGGCCCCGGCCGGGGGGCGGGCGCCGGCGGCTTTGGTGACTCTAGATAACCTCGGGCCGATCGCACGCCCCCCGTGGCGGCGACGACCCATTCGAACGTCTGCCCTATCAACTTTCGATGGTAGTCGCCGTGCCTACCATGGTGACCACGGGTGACGGGGAATCAGGGTTCGATTCCGGAGAGGGAGCCTGAGAAACGGCTACCACATCCAAGGAAGGCAGCAGG-digoxin	Sangon Biotech (Shanghai) Co., Ltd.

Table S7. Sequence of siRNA against specific target in this study

siRNA names	Sequences
sicircDLC1 sense	5’-UGUUUUAAGGGUAAUGCAATT-3’
sicircDLC1 anti-sense	5’-UUGCAUUACCCUUAAAACATT-3’
siDHX9 sense	5’-CUGAUCACAACAGGAGCUUTT-3’
siDHX9 anti-sense	5’-AAGCUCCUGUUGUGAUCAGTT-3’
siHuR-1 sense	5’-GGUUUGGCUUUGUGACCAUTT-3’
siHuR-1 anti-sense	5’-AUGGUCACAAAGCCAAACCTT-3’
siHuR-2 sense	5'-GAACGAAUUUGAUCGUCAATT-3'
siHuR-2 anti-sense	5'-UUGACGAUCAAAUUCGUUCTT-3'
siMMP1-1 sense	5'-GGAAUUCUUUGGGCUGAAATT-3'
siMMP1-1 anti-sense	5'-UUUCAGCCCAAAGAAUUCCTT-3'
siMMP1-2 sense	5'-CUAGAACUGUGAAGCAUAUTT-3'
siMMP1-2 anti-sense	5'-AUAUGCUUCACAGUUCUAGTT-3'
Control siRNA sense	5'-UUCUCCGAACGUGUCACGUTT-3'
Control siRNA anti-sense	5'-ACGUGACACGUUCGGAGAATT-3'
siKIAA1429-1 sense	5’-CCAUCAUCUUUAGACCUAATT-3’
siKIAA1429-1 anti-sense	5’-UUAGGUCUAAAGAUGAUGGTT-3’
siKIAA1429-2 sense	5'-CCUUACGUGUUCUCUGUAATT-3'
siKIAA1429-2 anti-sense	5'-UUACAGAGAACACGUAAGGTT-3'
